# Deep Learning-Based 30-Day Mortality Prediction in Critically Ill Bone and Bone Marrow Metastasis Patients: A Multicenter Retrospective Cohort Study

**DOI:** 10.3390/curroncol32100533

**Published:** 2025-09-24

**Authors:** Yixi Wang, Lintao Xia, Yuqiao Tang, Wenzhe Li, Jian Cui, Xinkai Luo, Hongyuan Jiang, Yuqian Li

**Affiliations:** 1 Department of Minimally Invasive Spine and Precision Orthopedics, The First Affiliated Hospital of Xinjiang Medical University, Urumqi 830054, China; wangyixi@stu.xjmu.edu.cn (Y.W.); 15798001657@163.com (X.L.); 2 State Key Laboratory of Pathogenesis, Prevention and Treatment of High Incidence Diseases in Central Asia, Xinjiang Medical University, Urumqi 830054, China; 13062801628@163.com; 3 Institute of Interdisciplinary Integrative Medicine Research, Shanghai University of Traditional Chinese Medicine, Shanghai 201203, China; 22024562@shutcm.edu.cn; 4 The Institute for Experiential AI Northeastern University, Boston, MA 02110, USA; tang.yuqi@northeastern.edu; 5 The College of Science, Northeastern University, Boston, MA 02115, USA; 6 Department of Critical Care Medicine, The First Affiliated Hospital of Xinjiang Medical University, Urumqi 830054, China; cxsfover1993@126.com; 7 Department of Bone Tumor, The First Affiliated Hospital of Xinjiang Medical University, Urumqi 830054, China; j1913972820@outlook.com; 8 Department of Anesthesiology, The First Affiliated Hospital of Xinjiang Medical University, Urumqi 830054, China

**Keywords:** bone and bone marrow metastasis, deep learning, prognosis, critical illness, risk stratification, 30-day mortality

## Abstract

Bone and bone marrow Metastasis (BBM) are life-threatening complications of advanced malignancies, frequently requiring intensive care and associated with high short-term mortality. However, prognostic tools specifically tailored to critically ill BBM patients are limited. This multicenter cohort study aimed to develop and validate deep learning models for predicting 30-day mortality using ICU data from MIMIC-IV, eICU-CRD, and the First Affiliated Hospital of Xinjiang Medical University. After univariate screening, XGBoost-Boruta and Lasso regression identified 11 key clinical features within 24 h of ICU admission. Thirteen deep learning models were trained using five-fold cross-validation, and their performance was evaluated through AUC, average precision, calibration, and decision curves. TabNet achieved the best internal performance (AUC 0.878; AP 0.940) and maintained strong discrimination in both same-region (eICU: AUC 0.840; AP 0.932) and cross-regional (Xinjiang: AUC 0.831; Accuracy 80.5%) validation. SHAP and attention-based interpretability analyses consistently identified SOFA, serum calcium, and albumin as dominant predictors. A TabNet-based online calculator was subsequently deployed to enable bedside mortality risk estimation. In conclusion, TabNet demonstrates potential as an accurate and interpretable tool for early mortality risk stratification in critically ill BBM patients, offering support for more timely and individualized decision-making in BBM-related critical care.

## Simple Summary

Patients with cancer that spreads to the bones and bone marrow often become critically ill and face a high risk of death, which makes reliable prognostic tools essential for determining whether intensive care should prioritize aggressive treatment or hospice approaches, yet existing scoring systems remain limited. This multicenter study used data from hospitals in the United States and China, incorporating routinely collected clinical information from the first day of intensive care to construct advanced computer models for predicting 30-day survival, and demonstrated that TabNet provided the most accurate and consistent performance across diverse cohorts, with its deployment as an online calculator enabling early and transparent risk stratification that supports timely clinical decision-making. These findings highlight the potential of deep-learning model to improve prognostic evaluation in oncologic critical care and to inform future research directions and policy strategies aimed at optimizing outcomes for patients with advanced cancer.

## 1. Introduction

Bone and bone marrow Metastasis (BBM) represents a devastating complication of advanced malignancies, constituting the third most frequent site of tumor dissemination following the lungs and liver [1]. Affected patients often experience rapid clinical deterioration characterized by intractable bone pain, pathological fractures, spinal cord compression, hematopoietic dysfunction, and hypercalcemia [2]. Despite significant advances in oncologic therapies, patients with BBM requiring intensive care unit (ICU) admission continue to face poor prognoses, with median survival often limited to only a few months [3]. In this context, accurate estimation of survival time plays a pivotal role in therapeutic decision-making in BBM patients, as a predicted life expectancy of less than one to three months typically warrants prioritization of palliative care and symptom management, whereas longer survival projections may support the consideration of orthopedic interventions or localized radiotherapy aimed at preserving functional status and improving quality of life [4,5,6,7]. Consequently, precise short-term mortality risk stratification is essential for optimizing treatment intensity, allocating critical care resources effectively, and facilitating informed discussions between clinicians and patients.

Nevertheless, practical tools for short-term prognostic assessment in critically ill BBM patients remain scarce. Despite efforts using conventional scores and machine learning approaches, current prognostic models inadequately address the clinical complexity of critically ill BBM patients, with limited robustness and bedside utility [8,9,10,11]. Deep learning (DL) has rapidly advanced in medical artificial intelligence, becoming a key tool for complex clinical prediction tasks [12]. Through multilayer neural networks, it automatically extracts high-dimensional features, captures nonlinear relationships, and identifies latent patterns, supporting applications in tumor diagnosis, risk stratification, and prognostic prediction [13]. Building on these advances, Thillai et al. [14] and Barnett et al. [15] leveraged DL for prognostic prediction and electroencephalogram pattern classification in critical care, respectively, both yielding substantial improvements in diagnostic accuracy and clinical utility.

To address the limitations of existing prognostic models and to develop a clinically deployable system for 30-day mortality prediction in critically ill BBM patients, this study systematically evaluated 13 cutting-edge DL architectures using real-world ICU data. The models included multilayer perceptron (MLP) [16,17], convolutional neural network (CNN) [18], DeepLearning [19], deep generalized linear model (Deep-GLM) [20], deep logistic regression (DLR) [21], Transformer [22], TabNet [23], generalized additive model neural network (GAM-NN) [24,25], graph neural network (GNN) [26], generative adversarial network (GAN) [27], Deep-Kernel [28], quantum neural network (QNN) [29], and Triplet Network [30,31]. By targeting critically ill BBM patients irrespective of primary tumor type, the study aimed to meet the urgent need for accurate risk stratification in highly heterogeneous and high-risk ICU BBM populations and to advance the clinical integration of DL models in oncologic critical care.

## 2. Materials and Methods

### 2.1. Data Sources

**Figure 1:**
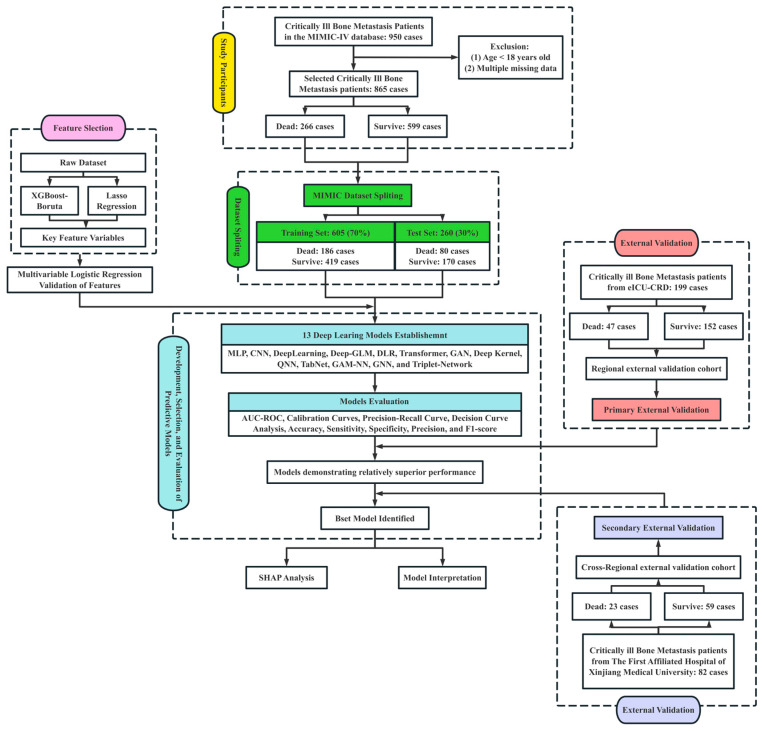
Study flowchart illustrating feature selection, model development, evaluation, and external validation processes. A total of 950 critically ill bone metastasis patients were initially identified from the MIMIC-IV database. After excluding patients aged under 18 years and those with multiple missing data points, 865 cases were included, comprising 266 deaths and 599 survivors. The dataset was randomly split into a training cohort (n = 605, 70%) and a test cohort (n = 260, 30%). Feature selection was performed using XGBoost-Boruta and Lasso regression, followed by multivariable logistic regression validation. Thirteen DL models were established, including MLP, CNN, DeepLearning, Deep-GLM, DLR, Transformer, GAN, Deep Kernel, QNN, TabNet, GAM-NN, GNN, and Triplet-Network. Model performance was evaluated using AUC-ROC, calibration curves, precision-recall curves, decision curve analysis, sensitivity, specificity, precision, and F1-score. The best-performing model underwent further SHAP analysis for model interpretation. External validation was conducted using two independent cohorts: a regional validation cohort from the eICU-CRD (n = 199) and a cross-regional validation cohort from The First Affiliated Hospital of Xinjiang Medical University (n = 82).

DL models for 30-day mortality prediction in critically ill BBM patients were constructed using the MIMIC-IV (Medical Information Mart for Intensive Care IV) v3.1 database (https://physionet.org/content/mimiciv/, accessed on 29 March 2025) [32]. To evaluate the model’s generalizability across healthcare systems, external validation was conducted using two independent cohorts: the eICU-CRD (eICU Collaborative Research Database) v2.0 [33], accessed on 2 April 2025, serving as a same-region external validation set, and a cohort from the First Affiliated Hospital of Xinjiang Medical University, serving as a cross-regional external validation set. Access to MIMIC-IV and eICU-CRD was obtained through PhysioNet credentialing (ID: 57264471), with all data usage adhering to PhysioNet’s Data Use Agreement and corresponding ethical standards. The ethics committee approved the use of institutional data from the First Affiliated Hospital of Xinjiang Medical University (K202404-45) (Figure 1).

### 2.2. Study Population and Data Extraction

Critically ill BBM patients were identified from the MIMIC-IV database based on ICD-9 and ICD-10 diagnostic codes, excluding individuals younger than 18 years or with substantial missing data. For patients with multiple ICU admissions due to BBM, only the first ICU admission was retained to minimize duplication bias, and data extraction was performed using Navicat Premium 15 (PremiumSoft CyberTech Ltd., Hong Kong, China). External validation cohorts were assembled by applying identical extraction criteria to the eICU-CRD and by manual abstraction from the electronic medical record system of the First Affiliated Hospital of Xinjiang Medical University. For each patient, 30-day survival status and survival time were recorded as outcomes, whereas predictor variables were restricted to those collected within the first 24 h of ICU admission, including demographic characteristics, comorbidities, rating system scores, vital signs, and laboratory test results for model development.

### 2.3. Data Preprocessing and Feature Selection

Variables with more than 30% missingness were excluded, while remaining missing values were imputed using Multiple Imputation by Chained Equations (MICE) to generate five complete datasets, and results were pooled using the “mice” package in R. Univariable analyses identified features with significant intergroup differences, and among clinically related variables concurrently significant, the most informative indicator was selected based on expert clinical judgment. Features identified through univariable screening were further refined using XGBoost-Boruta and Lasso regression to reduce redundancy and improve model parsimony [34], with chord diagram analysis confirming the absence of significant collinearity among the retained variables. The final feature set was validated via multivariable logistic regression across combined cohorts (MIMIC-IV, eICU-CRD, and the First Affiliated Hospital of Xinjiang Medical University) to confirm their independent prognostic relevance.

### 2.4. Handling Class Imbalance

In light of previous studies reporting an approximately 30% 30-day mortality rate among critically ill BBM patients [11], a mild class imbalance between survivors and non-survivors was anticipated. Considering the limited degree of imbalance, sample weighting and the Focal Loss function were incorporated during model development to preserve the original data distribution while enhancing sensitivity to high-risk patients, thereby improving the precision and clinical applicability of short-term prognostic predictions [35].

### 2.5. Statistical Analysis and Model Development

Categorical variables were reported as counts and percentages, and continuous variables as means with interquartile ranges (IQRs). Chi-square tests were used for categorical variables with expected frequencies above five, while Fisher’s exact tests were applied otherwise. The Wilcoxon rank-sum test was employed for continuous variables not meeting normality assumptions.

The development of the predictive model adhered to the TRIPOD (Transparent Reporting of a Multivariable Prediction Model for Individual Prognosis or Diagnosis) guidelines to ensure rigorous methodological transparency and reliability [36], with the TRIPOD-AI checklist provided in the Supplementary Materials. Thirteen DL models, including MLP (Feed-forward Neural Network) [16,17], CNN (LeNet-5-Style) [18], DeepLearning (H_2_O) [19], Deep-GLM [20], DLR [21], Transformer (Feature Tokenization) [22], TabNet (Supervised) [23], GAM-NN [24,25], GNN (Kipf–Welling) [26], GAN (Semi-Supervised and Feature Matching) [27], Deep-Kernel (SVGP, ARD-RBF, Bernoulli) [28], QNN (Variational Quantum Classifier) [29], and Triplet Network (Siamese, Margin Triplet Loss) [30,31], were developed using the MIMIC-IV cohort to predict 30-day mortality. A set of traditional machine learning models, including Logistic Regression (LR), Random Forest (RF), Support Vector Machine (SVM), K-Nearest Neighbors (KNN), Gradient Boosting (GB), AdaBoost, XGBoost, LightGBM, and CatBoost, was also developed to serve as comparators to the deep learning models. The MIMIC-IV dataset was randomly split into training and internal validation sets (7:3), and all models (both traditional and deep-learning) were developed under a unified framework with systematic hyperparameter tuning and five-fold cross-validation to ensure methodological consistency, optimize robustness, and mitigate overfitting. The core code for the thirteen models is available at https://github.com/Numwyx/Bone-and-Bone-Marrow-Metastasis-Model-Code (accessed on 9 September 2025), with additional details provided in Supplementary Material S1.

Model performance was evaluated through receiver operating characteristic (ROC) curves with the area under the curve (AUC) as a measure of discrimination, calibration plots, decision curve analysis, and precision–recall (PR) curves with the corresponding average precision (AP) used to summarize performance under class imbalance. Binary classification was based on a probability threshold of 0.5, and accuracy, sensitivity, specificity, precision, and F1 scores were calculated accordingly. The optimal model was selected based on performance in the internal and same-region external validation cohorts, interpreted through SHapley Additive exPlanations (SHAP), and further evaluated for generalizability using cross-regional validation data. Upon confirming consistent and stable performance, the model was deployed as an online tool to support bedside risk assessment and clinical decision-making.

All statistical analyses, model development, and SHAP interpretability were conducted in R 4.3.3 (R Foundation for Statistical Computing, Vienna, Austria), with cross-platform computation enabled through the “reticulate” package interfacing with Python 3.9.21 (Python Software Foundation, Wilmington, DE, USA). Statistical significance was defined as a two-sided *p* value less than 0.05.

## 3. Results

### 3.1. Study Population and Baseline Characteristics

According to the predefined inclusion and exclusion criteria, a total of 865 eligible critically ill BBM patients were identified from the MIMIC-IV database (See Figure 1 for the detailed screening process), of whom 266 died within 30 days. Univariable analyses stratified by 30-day survival status revealed significant differences across multiple clinical variables between survivors and non-survivors, as detailed in Table 1. In the same-region external validation cohort (eICU-CRD), 152 patients survived and 47 died within 30 days (Supplementary Table S1), whereas in the cross-region external validation cohort (The First Affiliated Hospital of Xinjiang Medical University), 59 patients survived and 23 died within 30 days (Supplementary Table S2).

### 3.2. Feature Selection and Validation

**Figure 2:**
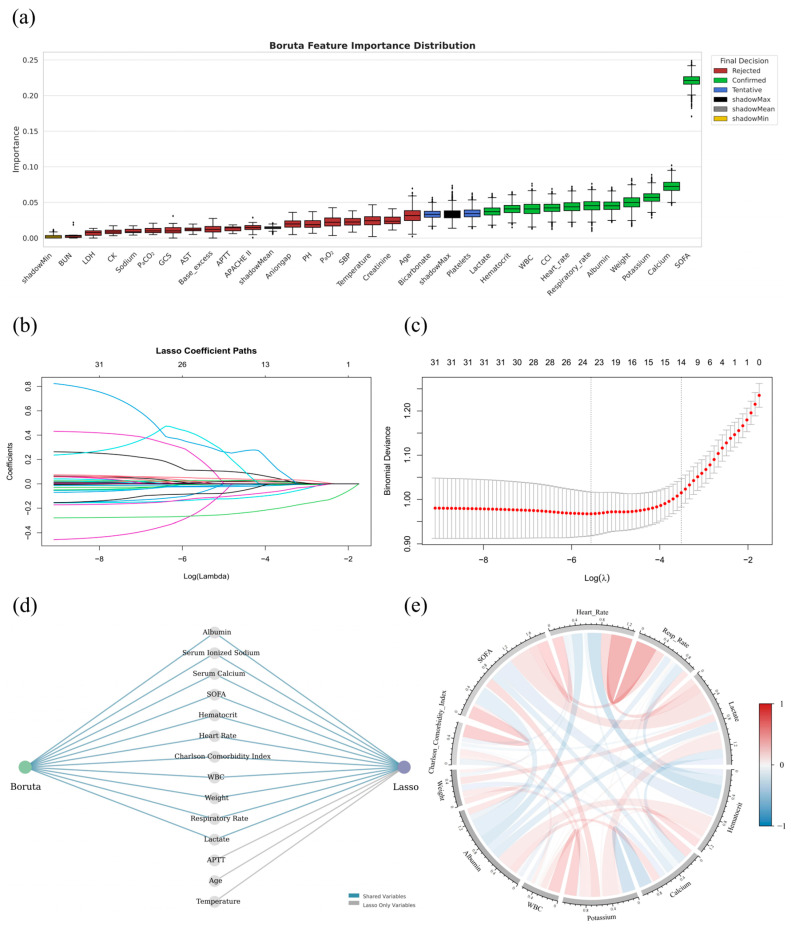
Feature selection and correlation analysis of key predictors associated with 30-day mortality in critically ill BBM patients. (**a**): Distribution of feature importance scores obtained from the Boruta algorithm based on the MIMIC-IV dataset. Features are categorized into Confirmed (green), Tentative (blue), and Rejected (red) according to their relevance, with shadow features (shadowMin, shadowMean, shadowMax) used as baseline references. (**b**): Coefficient trajectories for all variables across a series of penalization strengths (log-transformed λ values) in Lasso regression. Each line represents a variable, with non-zero coefficients progressively eliminated as the penalty increases. (**c**): Ten-fold cross-validation results for Lasso regression, showing the mean binomial deviance across a range of log(λ) values. The λ corresponding to the minimum deviance (λ_min) and the λ within one standard error of the minimum (λ_1SE) are indicated by vertical dashed lines. To optimize model simplicity and generalizability, λ_1SE was selected, resulting in the retention of 14 non-zero feature coefficients; (**d**): Integration of Boruta and Lasso feature selection outcomes. Variables selected by both methods are identified as shared features (blue lines), whereas features selected uniquely by Lasso are marked separately (gray lines); (**e**): Chord diagram depicting pairwise Pearson correlation coefficients among the selected key variables. The width and color of the chords represent the strength and direction of correlations, with red indicating positive associations and blue indicating negative associations.

Among the 37 variables significantly associated with 30-day mortality, several captured overlapping physiological functions, such as systolic and diastolic blood pressure within the cardiovascular system. Recognizing the high degree of correlation among these functionally similar features, 30 representative variables were further selected through clinically guided prioritization to reduce redundancy while maintaining interpretability and served as candidate inputs for subsequent feature selection. Key features were identified through XGBoost-Boruta (Figure 2a) and Lasso regression (Figure 2b,c), yielding 11 and 14 variables, respectively, with substantial concordance. The final model incorporated weight, Charlson Comorbidity Index (CCI), SOFA score, heart rate, respiratory rate, lactate, hematocrit, serum ionized calcium (hereinafter referred to as serum calcium), serum sodium, white blood cell count (WBC), and albumin (Figure 2d). Collinearity diagnostics confirmed minimal interdependence, supporting their inclusion as distinct predictors (Figure 2e).

Although some predictors such as serum potassium, serum calcium, and WBC counts are less frequently emphasized in general ICU mortality models, their inclusion in the final model is supported by evidence specific to critically ill patients with bone and bone marrow metastases, in which potassium abnormalities arising from renal impairment, tumor lysis, or treatment-related electrolyte shifts have been associated with increased short-term mortality [37,38]. Disturbances in calcium homeostasis, particularly hypocalcemia that occurs secondary to osteolytic activity or antiresorptive therapy, have been linked to adverse outcomes [39], while abnormal WBC counts that indicate bone marrow infiltration, systemic inflammation, or chemotherapy-induced myelosuppression carry significant prognostic implications in this population [40], thereby capturing metabolic, skeletal, and hematologic pathways that are not fully represented by more conventional predictors yet are highly relevant to mortality risk.

To assess the selected features’ independent prognostic value, multivariable logistic regression was conducted across the combined MIMIC-IV, eICU-CRD, and Xinjiang cohorts. All variables remained statistically significant in the multivariable model, confirming their independent predictive relevance (Table 2). The Hosmer–Lemeshow test yielded a χ^2^ of 3.77 (*p* = 0.877), indicating excellent calibration and no significant deviation between predicted and observed outcomes, further supporting the robustness of feature selection.

### 3.3. Comparative Performance of DL Models

**Figure 3:**
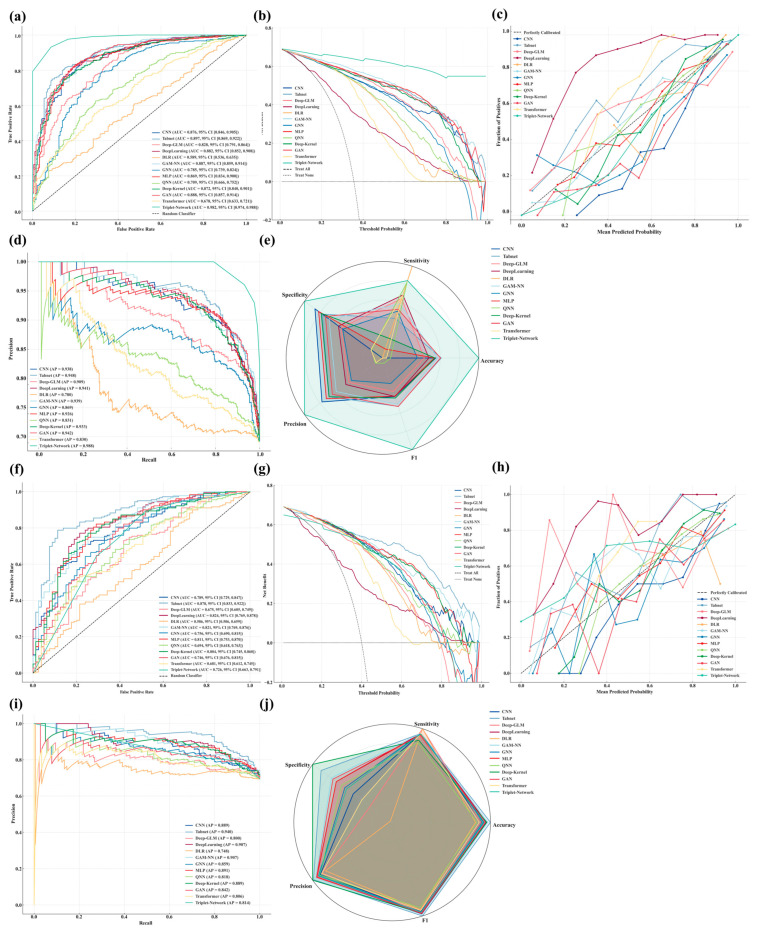
Comprehensive evaluation of 13 DL models for predicting 30-day mortality in critically ill BBM patients. (Train: (**a**), Test: (**f**)): ROC curves with AUC and 95% confidence intervals were plotted to assess model discrimination in the training and test sets; (Train: (**b**), Test: (**g**)): Decision curve analysis was conducted to evaluate clinical net benefit across a range of threshold probabilities; (Train: (**c**), Test: (**h**)): Calibration curves were generated to examine the agreement between predicted and observed risks; (Train: (**d**), Test: (**i**)): Precision-recall curves were used to assess performance under class imbalance; (Train: (**e**), Test: (**j**)): Radar plots summarizing normalized accuracy, sensitivity, specificity, precision, and F1-score were constructed to enable cross-model comparisons.

Traditional non-deep learning models, including logistic regression, support vector machines, k-nearest neighbors, random forests, and gradient boosting variants (e.g., XGBoost, CatBoost, LightGBM), were unable to achieve accurate prediction of 30-day mortality in critically ill patients with bone and bone marrow metastases, showing diminished discrimination, insufficient calibration, and unstable clinical net benefit in both training and test evaluations (Supplementary Material S2). In contrast, deep learning–based models demonstrated superior predictive performance across multiple metrics, providing better discrimination, calibration, and clinical utility. The final optimized hyperparameter configurations for each model are presented in Supplementary Table S3. Figure 3a–j show Model performance in the training (a–e) and internal validation cohorts (f–j), including ROC curves (a,f), decision curve analyses (b,g), calibration curves (c,h), PR curves (d,i), and radar plots summarizing classification metrics (e,j). TabNet demonstrated the most stable performance across discrimination, precision, calibration, and clinical utility, with minimal declines in AUC (Train: 0.897; Test: 0.878) and AP (Train: 0.948; Test: 0.940). Predicted risks aligned closely with observed outcomes, and net benefit remained optimal across all thresholds. DeepLearning, GAM-NN, MLP, and Deep-Kernel also showed strong generalizability, with small performance losses, well-calibrated predictions, and consistent decision-analytic value. Radar plots confirmed balanced classification metrics across these five models, supporting their use in high-risk settings. Triplet-Network achieved the highest AUC and AP in training (0.982 and 0.988, respectively) and outperformed others in decision analysis but declined sharply in testing (AUC 0.726; AP 0.814), indicating overfitting. CNN, GNN, and GAN exhibited moderate declines in test performance and did not demonstrate distinct advantages over other models, although their overall utility remained acceptable. Deep-GLM, QNN, Transformer, and DLR underperformed across all metrics, with poor calibration and negligible clinical benefit. Radar plots ranked DLR and Transformer lowest in all classification measures, suggesting limited applicability.

### 3.4. Same-Region External Validation

Given their strong performance in MIMIC-IV training and internal validation, TabNet, DeepLearning, GAM-NN, MLP, and Deep-Kernel were further evaluated using an external validation cohort from the same healthcare region (eICU-CRD) to assess generalizability. The clinical characteristics of critically ill BBM patients in the eICU-CRD cohort, including 152 who survived and 47 who died within 30 days, are summarized in Supplementary Table S1.

**Figure 4:**
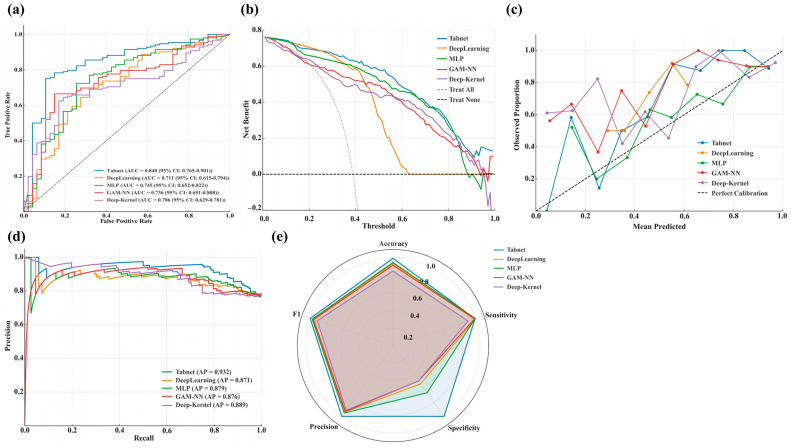
Regional external validation of selected high-performing DL models using the eICU-CRD dataset. (**a**): ROC curves displaying the true positive rate against the false positive rate, with AUC and 95% confidence intervals reported for each model; (**b**): Decision curves presenting the net benefit across a range of threshold probabilities; (**c**): Calibration curves plotting observed proportions versus mean predicted probabilities; (**d**): PR curves showing the relationship between precision and recall; (**e**): Radar charts presenting normalized values of accuracy, sensitivity, specificity, precision, and F1-score for comparative model assessment.

TabNet demonstrated the most robust external performance across all key domains, with an AUC of 0.840 and an AP of 0.932, reflecting only minor declines from its internal validation set values in MIMIC-IV (AUC 0.878 and AP 0.940). It also demonstrated the highest net clinical benefit across all threshold probabilities and maintained excellent calibration, with predicted risks closely aligned with observed outcomes. In addition, radar plots confirmed stable and balanced classification performance across accuracy, sensitivity, specificity, precision, and F1 score, supporting its suitability for high-risk clinical settings. By contrast, DeepLearning, GAM-NN, MLP, and Deep-Kernel showed notable reductions in discrimination and precision, accompanied by degraded calibration and limited clinical benefit, indicating insufficient generalizability and leading to their exclusion from further evaluation. Figure 4a–e illustrates external validation results in the eICU-CRD, including ROC curves (a), decision curves (b), calibration plots (c), precision-recall curves (d), and radar charts across key performance dimensions (e).

### 3.5. Sequential Attention Mechanism of TabNet

**Figure 5:**
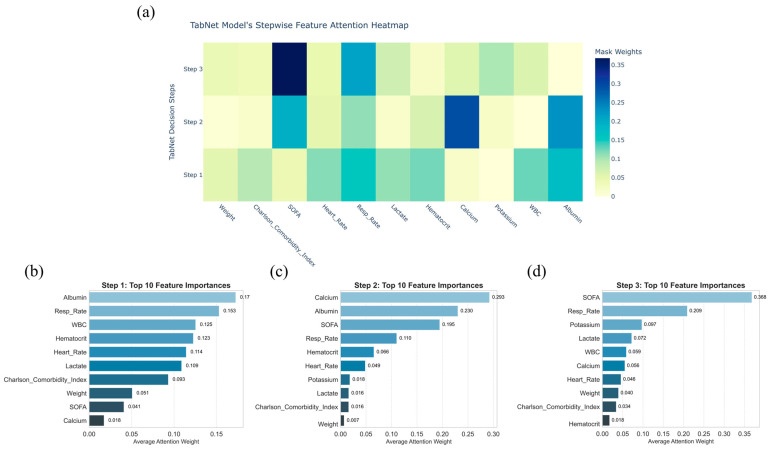
Stepwise feature attention analysis of the TabNet model for predicting critically ill bone metastasis patients’ 30-day mortality. (**a**): Heatmap showing the distribution of feature attention weights across decision steps 1 to 3; (**b**–**d**): bar plots displaying the top 10 features ranked by average attention weights at each corresponding decision step.

The sequential attention mechanism of TabNet, which dynamically selects and reweights features at each decision step to provide an interpretable trajectory of how the model arrives at its predictions, offers a dynamic and transparent view of its decision process in predicting 30-day mortality among critically ill BBM patients (Figure 5a). In the first step, attention was concentrated on features indicative of nutritional status, inflammation, and vital signs, notably albumin, respiratory rate, and WBC (Figure 5b). Subsequent steps shifted focus toward organ dysfunction and metabolic imbalance, with serum calcium, albumin, and SOFA score emerging as key variables (Figure 5c). By the final stage, the SOFA score dominated, followed by respiratory rate and serum potassium, reflecting progressive weighting toward indicators of organ failure (Figure 5d). This hierarchical reallocation of feature importance, visualized in the attention heatmap, aligns with clinical reasoning patterns in critical care prognosis.

### 3.6. SHAP Analysis

**Figure 6:**
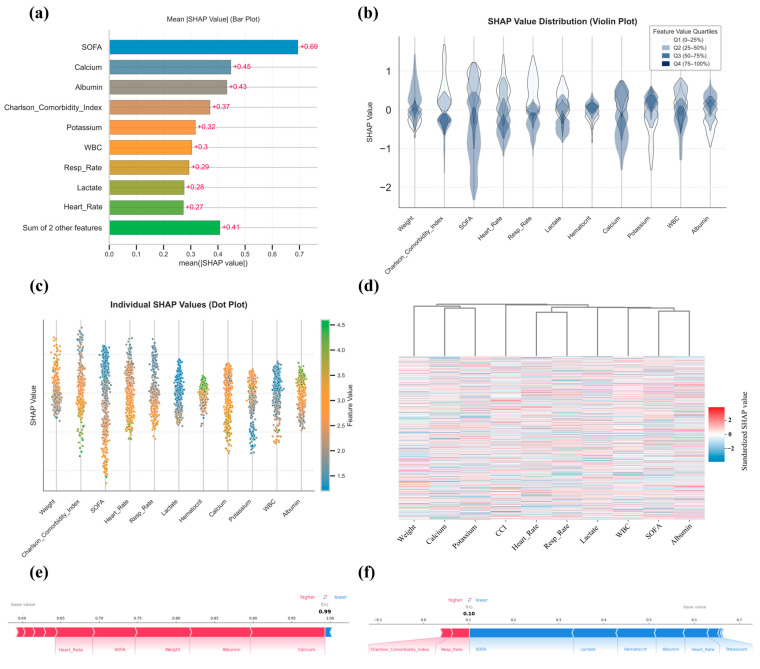
SHAP-based interpretability analysis of the TabNet model. (**a**): Bar plot displaying the mean absolute SHAP values for top-ranked features; (**b**): Violin plots showing the distribution of SHAP values stratified by feature value quartiles; (**c**) Beeswarm plot illustrating SHAP value distributions across features, with point colors indicating original feature values; (**d**): Heatmap with hierarchical clustering based on standardized individual SHAP values across patients; (**e**,**f**): SHAP force plots visualizing individual prediction explanations for representative survival (**e**) and death (**f**) cases, respectively.

To further dissect the predictive mechanism of TabNet, a multi-level SHAP (SHapley Additive exPlanations) analysis was performed, a game-theoretic approach that quantifies the contribution of each feature to model predictions while providing both global importance rankings and case-specific explanations. Mean absolute SHAP values ranked SOFA score, serum calcium, albumin, CCI, and serum potassium as the most influential features (Figure 6a). The associations between the five top SHAP-ranked variables (SOFA score, serum calcium, serum albumin, Charlson Comorbidity Index, and serum potassium) and mortality were assessed using the Mann–Whitney U test, point-biserial correlation, and Cliff’s δ, with violin plots depicting the distributions for the survival and death groups. All five variables showed statistically significant differences between groups, with effect sizes ranging from small (potassium, calcium) to large (SOFA score), indicating varying degrees of clinical relevance, with detailed results provided in Supplementary Material S3. Violin plots showed inter-individual variability, with wide SHAP distributions for weight, CCI, SOFA score, heart rate, and respiratory rate, reflecting heterogeneous impacts, whereas serum potassium and hematocrit showed more stable contributions (Figure 6b). Beeswarm plots revealed directional associations, with lower weight, elevated CCI, higher SOFA score, tachycardia, increased respiratory rate, elevated lactate, hypocalcemia, low hematocrit, and leukocytosis all linked to greater mortality risk, whereas higher albumin was protective (Figure 6c). SHAP heatmaps displayed the distribution of feature contributions across individuals, highlighting the discriminative value of weight, serum calcium, serum potassium, SOFA, and albumin (Figure 6d). Force plots from two representative cases (Death: Figure 6e, Survival: Figure 6f) further illustrate patient-specific decision pathways.

### 3.7. Cross-Region External Validation

**Figure 7:**
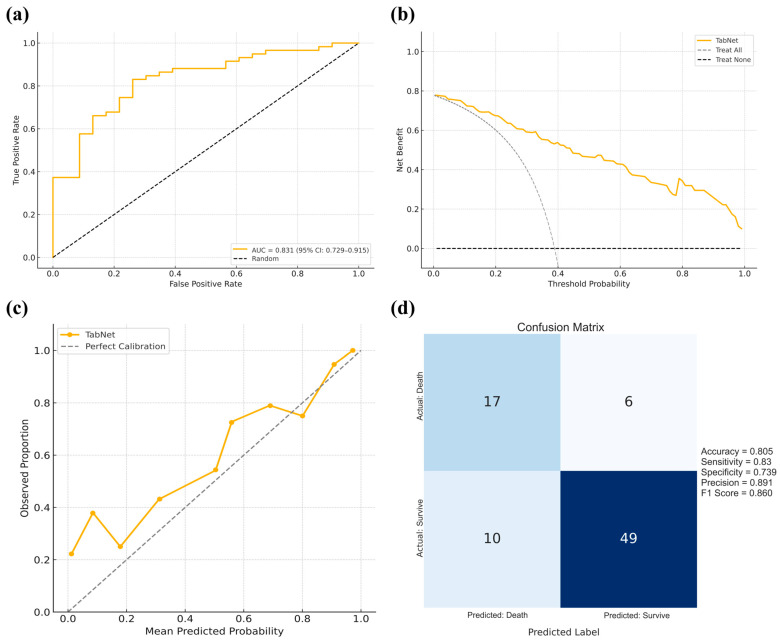
Cross-regional external validation of the TabNet model using data from the First Affiliated Hospital of Xinjiang Medical University. (**a**): ROC curve with the AUC and 95% confidence interval; (**b**): Decision curve analysis comparing the net clinical benefit of the TabNet model against the treat-all and treat-none strategies across threshold probabilities; (**c**): Calibration curve showing the agreement between predicted and observed outcomes; (**d**) Confusion matrix summarizing model performance, with corresponding accuracy, sensitivity, specificity, precision, and F1-score.3.7 Cross-region external validation.

In the cross-region external validation cohort from the First Affiliated Hospital of Xinjiang Medical University, which included 59 patients who survived and 23 who died within 30 days, TabNet demonstrated consistent performance, with an AUC of 0.831 (95% CI: 0.729–0.915), indicating strong discriminative power (Figure 7a). Decision curve analysis revealed superior net clinical benefit across most thresholds compared with treat-all or treat-none strategies (Figure 7b), while calibration curves showed excellent agreement between predicted and observed risks (Figure 7c). The model achieved an accuracy of 80.5%, sensitivity of 83.0%, specificity of 73.9%, precision of 89.1%, and an F1 score of 86.0%, reflecting well-balanced classification (Figure 7d). These results support the Tabnet model’s generalizability and clinical applicability across geographically diverse populations. The clinical characteristics of critically ill BBM patients in the First Affiliated Hospital of Xinjiang Medical University cohort are summarized in Supplementary Table S2.

### 3.8. Robustness and Generalizability of Key Predictors

**Figure 8:**
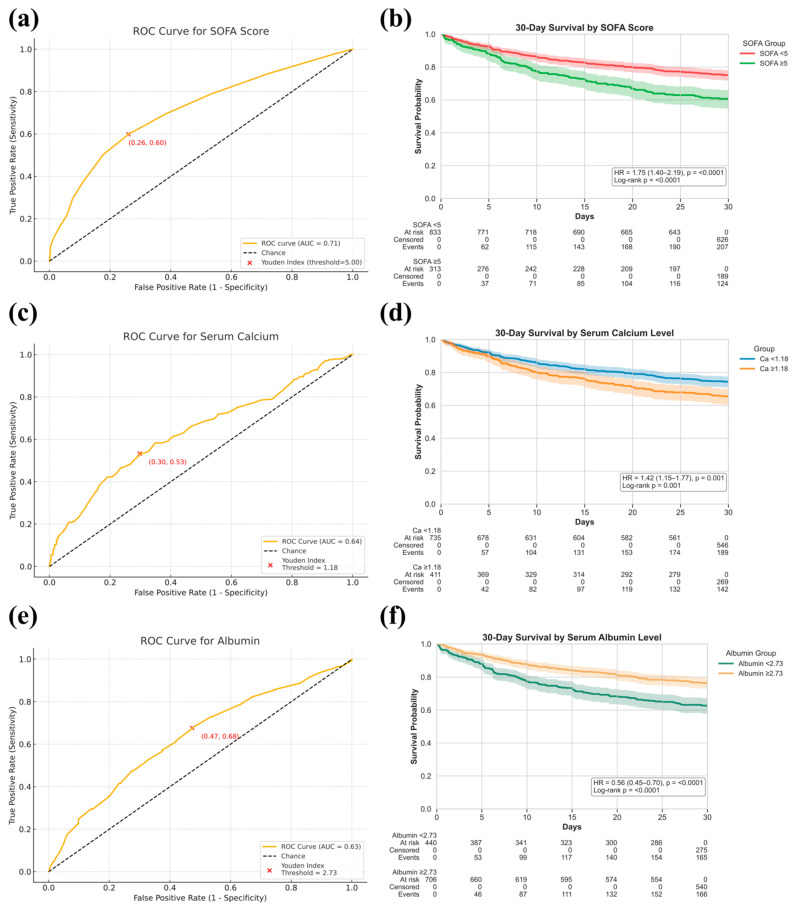
Prognostic value of SOFA score, serum calcium, and serum albumin levels for 30-day mortality prediction in critically ill bone metastasis patients. (**a**,**c**,**e**): ROC curves showing discrimination performance for SOFA score (**a**), serum calcium (**c**), and serum albumin (**e**), with AUCs reported and optimal thresholds determined by the Youden Index; (**b**,**d**,**f**): Kaplan–Meier survival curves comparing 30-day survival probabilities between stratified groups based on SOFA score ((**b**), <5 vs. ≥5), serum calcium ((**d**), <1.18 mmol/L vs. ≥1.18 mmol/L), and serum albumin ((**f**), <2.73 g/dL vs. ≥2.73 g/dL), with hazard ratios (HR) and log-rank *p*-values reported.

TabNet’s sequential feature selection and SHAP analysis consistently identified SOFA score, serum calcium, and albumin as dominant predictors of 30-day mortality. To evaluate their robustness across populations, a multicenter analysis was conducted using pooled ICU data from MIMIC-IV, eICU-CRD, and the First Affiliated Hospital of Xinjiang Medical University. In the combined cohort, ROC analysis demonstrated certain prognostic value for SOFA (Figure 8a, AUC 0.71, cutoff 5.00), serum calcium (Figure 8b, AUC 0.64, cutoff 1.18 mmol/L), and albumin (Figure 8c, AUC 0.63, cutoff 2.73 g/dL). Patients stratified by optimal thresholds showed significant survival differences in Kaplan–Meier analysis, confirming the stability of these markers across datasets (Figure 8d: SOFA; Figure 8e: Calcium; Figure 8f: Albumin).

**Figure 9:**
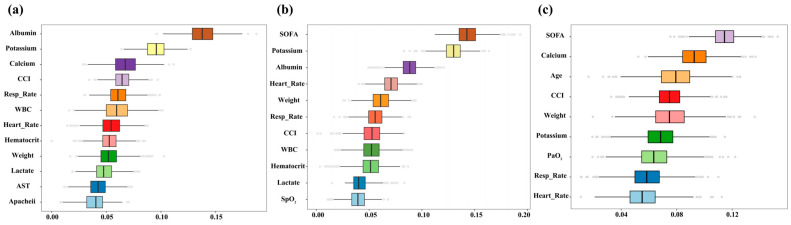
Feature selection results after excluding SOFA score, serum calcium, and serum albumin. (**a**): Boxplots displaying the distribution of feature importance scores after removing SOFA score; (**b**): Boxplots showing feature importance after excluding serum calcium; (**c**): Boxplots illustrating feature importance rankings following the exclusion of serum albumin.

In patients classified as high-risk based on SOFA score, serum calcium, or albumin levels, re-analysis after excluding each corresponding variable yielded feature selection results largely consistent with the initial feature selection (Figure 9a: excluding SOFA; Figure 9b: excluding Calcium; Figure 9c: excluding Albumin). Although SOFA, calcium, and albumin contributed most prominently, consistent signals from other predictors across datasets and strata indicate that model performance is driven by integrated clinical information rather than isolated variables, supporting its robustness and cross-population applicability.

### 3.9. Web Calculator

**Figure 10:**
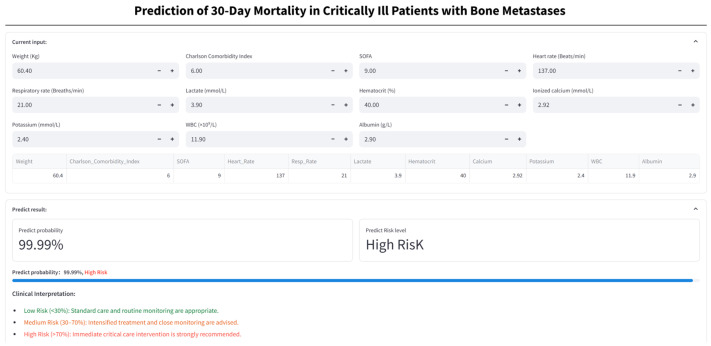
Web-based calculator for 30-day mortality risk in critically ill bone metastasis patients. Patient clinical variables are entered into the online calculator to generate the predicted probability of 30-day mortality and the corresponding risk stratification (low, medium, or high), and assist decision-making based on the assigned risk category.

A TabNet-based web calculator (https://numwyx-tabnetapp.streamlit.app/, accessed on 5 May 2025) was developed to facilitate rapid estimation of 30-day mortality risk in critically ill BBM patients through simple input of relevant clinical parameters (Figure 10).

## 4. Discussion

This study evaluated 30-day mortality in critically ill BBM patients using 13 DL models, among which TabNet demonstrated the highest predictive accuracy and generalizability, with consistently superior performance across datasets. As an interpretable attention-based architecture, TabNet enables end-to-end learning while simultaneously achieving feature sparsity and nonlinear representation, thereby eliminating the need for extensive feature engineering and balancing performance with transparency [23]. Its broad applicability in medical AI has been supported by prior studies, including Joseph et al., who reported 92.2% accuracy in early diabetes prediction [41], and Kita et al., who confirmed its efficacy in spinal tumor classification [42], further underscoring its reliability in clinical risk modeling.

The TabNet model was trained using 11 routinely available ICU variables, including weight, CCI, SOFA score, heart rate, respiratory rate, lactate, hematocrit, serum calcium, sodium, WBC, and albumin, which collectively reflect baseline physiology, organ dysfunction, and metabolic imbalance. Variable selection prioritized clinical accessibility and pathophysiological relevance, enhancing interpretability and translational potential without compromising model stability. Grounded in routinely available data, the framework enables scalable early risk stratification and personalized intervention for critically ill BBM patients at high short-term mortality risk.

Albumin emerged as a primary driver in the TabNet model’s initial decision layer, a finding corroborated by SHAP analysis, which associated lower levels with substantially increased 30-day mortality in critically ill BBM patients. As a well-established negative acute-phase reactant, serum albumin declines in response to systemic inflammation and physiological stress, reflecting diminished nutritional reserve and underlying metabolic or inflammatory burden [43]. Clinically, hypoalbuminemia has been consistently linked to adverse outcomes across a range of conditions, including cirrhosis, renal failure, major burns, and malignancies [44]. Ali et al. reported that each 1 g/dL decrease in preoperative albumin was associated with a 6.21-fold increase in one-year mortality in patients following proximal femoral metastasis resection [45], while Hsieh et al. also identified higher albumin levels as an independent predictor of prolonged survival in BBM patients [46]. These findings align with our results, where Kaplan–Meier analysis revealed significantly higher early mortality in the low-albumin subgroup, underscoring its value as a mechanistically grounded, stable, and clinically accessible biomarker for early risk stratification of critically ill BBM patients.

In the TabNet model developed in this study, serum calcium emerged as a key prognostic variable in the second decision step, with SHAP analysis confirming its strong association with 30-day mortality in critically ill BBM patients. Although studies have reported inconsistent associations between serum calcium levels and the risk of developing BBM [47,48], hypercalcemia is consistently recognized as a marker of poor prognosis once bone Metastasis are established [49]. Kaplan–Meier analysis in this study corroborated these findings, demonstrating significantly reduced short-term survival in patients with hypercalcemia. Mechanistically, elevated serum calcium reflects a profound disruption of skeletal homeostasis, characterized by tumor-induced suppression of osteoblasts, overactivation of osteoclasts, and remodeling niche dysfunction [50]. This pathological process occurs in both osteolytic Metastasis, such as multiple myeloma and breast cancer, where bone resorption predominates, and in osteoblastic lesions, typified by prostate cancer, where overexpression of parathyroid hormone-related protein promotes osteoblast precursor differentiation while concurrently driving osteoclastogenesis, sustaining high skeletal turnover despite radiographic sclerosis [51,52]. Notably, emerging studies show bone-metastatic tumors can, via self-reinforcing vicious cycles and calcium-sensing receptor upregulation, promote tumor proliferation and skeletal tropism, thereby accelerating metastatic progression [53,54]. Therefore, the onset of hypercalcemia in critically ill BBM patients warrants prompt clinical management to control calcium levels and limit skeletal resorption [55], with the intent to reduce metabolic complications, although its impact on survival outcomes has yet to be established.

This study offers the first comprehensive assessment of the SOFA score’s prognostic value in critically ill oncology patients. Within the TabNet model, SOFA consistently ranked among the most influential predictors across multiple decision steps, indicating its robust and stable contribution. Pre-model Boruta selection further prioritized SOFA with the highest importance score, and an independent AUC of 0.71 supported its discriminative capacity. While traditionally applied in general ICU mortality risk assessment, SOFA has gained increasing recognition in cancer-related critical illness [56]. Silvio et al. identified SOFA at admission as predictive of short-term mortality in tumor patients [57], and Marlou et al. found that SOFA scores exceeding 5.2 were associated with a markedly increased risk of mortality in end-stage cancer patients [58]. In this study, 30-day non-survivors had markedly higher SOFA scores than survivors (5.55 vs. 3.03), with survival analysis demonstrating a clear mortality gradient across SOFA strata, aligning with findings by Miao et al., who identified SOFA as an independent predictor of short-term mortality in critically ill BBM patients [11]. SHAP analysis also demonstrated that lower SOFA scores consistently aligned with favorable predictions, reflecting a stable and directionally concordant contribution to model outputs. Within this research, the SOFA score exhibited consistent prognostic utility for short-term mortality in BBM critical illness, with particularly strong discriminative performance in patients with multisystem failure or reduced physiological reserve. These findings underscore its value in oncologic risk stratification, where its integration into predictive frameworks may enhance sensitivity to organ impairment, improve model interpretability, and support individualized clinical decision-making in complex, high-risk populations.

Albumin, serum calcium, and SOFA score were central to the TabNet decision pathway. To evaluate the stability of feature selection, Boruta analysis was reapplied within high-risk subgroups defined by elevated SOFA scores, hypercalcemia, or hypoalbuminemia across the MIMIC-IV, eICU-CRD, and Xinjiang cohorts. The resulting features showed strong concordance with those derived from the unstratified MIMIC population, supporting the robustness and cross-cohort generalizability of the feature selection strategy. This consistency provides a foundation for further investigation of additional model-included features that may offer deeper mechanistic insights into short-term mortality among critically ill BBM patients.

Hypercalcemia-induced hypokalemia has been well-documented in BBM patients, with the phenomenon attributed to renal mechanisms involving activation of calcium-sensing receptors in the thick ascending limb, which suppress sodium–potassium–chloride cotransporter activity and promote kaliuresis, while concurrent volume contraction stimulates aldosterone release and further exacerbates potassium loss [59,60,61]. This study’s data revealed an inverse relationship between serum calcium and potassium levels, and SHAP analysis further identified hypokalemia as a marker of poor 30-day survival. These findings support a management strategy focused on volume optimization, calcium load reduction, and targeted electrolyte correction. Therapeutic regimens should avoid agents that worsen metabolic derangement and instead use dynamic monitoring to guide individualized fluid and potassium supplementation, with the goal of restoring homeostasis and improving clinical outcomes.

Compelling evidence identifies weight as a key prognostic determinant across malignancies, with lower weight linked to elevated mortality in colorectal and lung cancers [62,63], and a meta-analysis by Wen et al. confirms that both underweight and weight loss predict poorer survival [64]. In patients with bone Metastasis, this relationship is supported by Heish et al. [46] and reinforced by SHAP analysis within the TabNet model, which consistently recognized body weight as a stable and favorable predictor of short-term outcomes. As low weight often reflects diminished nutritional and physiological reserves, nutritional intervention in this population requires careful modulation [65]. Rapid repletion may trigger metabolic instability, particularly in those with impaired organ function, necessitating a strategy centered on physiologic tolerability and gradual restoration to improve nutritional status while minimizing systemic stress.

Extensive research has established that the CCI is positively correlated with mortality risk in critically ill patients, and, compared to acute physiology scores such as APACHE II, CCI more effectively reflects the long-term prognostic implications of baseline comorbidity burden [66]. Among BBM patients, elevated CCI has been associated with a spectrum of adverse clinical outcomes, including non-infectious complications, venous thromboembolism, increased short-term mortality following pathological fractures, and treatment-related death during palliative radiotherapy, collectively reinforcing its predictive relevance in this high-risk population [67,68,69,70]. In line with prior evidence, SHAP analysis identified lower CCI scores as consistently contributing to favorable survival predictions, further substantiating its independent value in short-term risk stratification.

Elevated lactate has been consistently associated with adverse outcomes in BBM patients, as demonstrated by Schwickert et al., who reported significant correlations between higher lactate levels and reduced overall and disease-free survival [71]. Beyond promoting angiogenesis, invasiveness, and immune evasion, lactate modulates metabolic pathways and acidifies the bone microenvironment, thereby facilitating tumor colonization and skeletal metastasis [72]. Following osseous involvement, lactate further accelerates bone resorption by enhancing osteoclast activity, exacerbating skeletal fragility, and increasing metastatic risk [73]. This pathophysiological profile aligns with SHAP analysis, which identified lower lactate levels as predictors of improved 30-day survival. These findings underscore lactate’s dual relevance as both a driver of metastatic progression and a potential biomarker for short-term prognostication in critically ill cancer patients.

Leukocyte levels have been closely linked to short-term outcomes in advanced malignancies [74], a relationship that also extends to BBM patients, as shown by Huang and Hsieh, who reported that lower WBC predicted better survival, a pattern subsequently reflected in the SHAP analysis [46,75]. This study similarly highlighted heart rate, respiratory rate, and hematocrit as relevant predictors, which, although not yet validated in the context of bone Metastasis, reflect underlying physiological stress or impaired perfusion in critically ill patients. Tachycardia and tachypnea, indicative of hypoxemia, systemic infection, or metabolic derangement, alongside hemoconcentration reflected by elevated hematocrit, may signal impending physiologic collapse if left uncorrected, thereby accelerating clinical deterioration and compromising short-term survival [76]. These routinely acquired parameters, though often overlooked, may therefore offer valuable prognostic insight in the BBM high-risk population and warrant greater attention in early risk stratification and supportive management.

This study is the first to systematically evaluate 13 forefront DL algorithms for predicting 30-day mortality in critically ill BBM patients with bone Metastasis, with the goal of providing quantitative support for short-term prognostic assessment while informing therapeutic intensity decisions, resource allocation, and clinical communication. Despite favorable model performance, several limitations merit consideration. First, the rarity of ICU-admitted BBM patients resulted in a limited sample size, potentially constraining variable inclusion and increasing overfitting risk. Nevertheless, the TabNet model maintained strong generalizability across two external validation cohorts, suggesting robust stability. Second, mild class imbalance was addressed through sample weighting and Focal Loss, which improved sensitivity for high-risk individuals without distorting the overall data structure. Third, to account for inevitable missingness in public datasets, multiple imputation using fully conditional specification under the MICE framework was employed, enhancing model reliability while preserving data completeness. Fourth, as a retrospective analysis, selection bias remains a concern, and the absence of certain clinically relevant variables not captured in the available datasets, such as cancer treatment status, tumor burden, and other disease-specific factors, may act as potential sources of unmeasured confounding, which could modestly influence risk estimation and external validity. Previous studies have demonstrated that treatment history, disease extent, and tumor burden are independent prognostic determinants in patients with bone metastases and critical illness, and omission of these variables may attenuate predictive accuracy and constrain model generalizability [77,78,79]. Future prospective studies should aim to incorporate these variables to improve model robustness and interpretability. Fifth, given the clinical relevance of the 90-day prognosis in BBM populations [80], future work may extend this framework to longer-term prediction to broaden its decision-support utility.

In addition to the aforementioned limitations, it is worth noting that variability in ICU admission thresholds and triage practices across datasets may introduce a degree of spectrum and case-mix bias that cannot be fully avoided. MIMIC-IV, the First Affiliated Hospital of Xinjiang Medical University, and eICU-CRD naturally reflect different healthcare environments with heterogeneous triage practices, resource availability, and intermediate care capacity, shaped by institutional policies and clinical standards. Such variability may lead to modest shifts in baseline mortality and in the distribution of physiologic traits recorded in the first 24 h, which in turn can influence model calibration and the apparent transportability of predictions across settings [81,82,83]. Methodological guidance recommends that prediction models be evaluated for calibration and transportability in case mixes similar to the intended use setting, with recalibration or updating applied if performance drifts across sites [84,85]. To address this, in the present study, variable definitions and time windows were carefully standardized across MIMIC-IV, eICU-CRD, and the local institutional cohort, which reduces potential bias, although some residual differences in admission timing are likely to persist, especially in cross-regional cohorts. In addition, the study incorporated multi-site external validation, explicitly using eICU-CRD as a same-region validation set to assess robustness under broadly comparable healthcare conditions, and the First Affiliated Hospital of Xinjiang Medical University as a cross-regional validation set to evaluate adaptability under distinct clinical environments. This dual validation approach provides a balanced assessment of both regional generalizability and cross-regional applicability. Future work may further harmonize ICU admission criteria across centers, capture pre-admission clinical trajectories, and perform site-specific recalibration to reduce residual bias, but the consistency of results across multiple datasets in this study suggests that the overall conclusions remain robust.

## 5. Conclusions

In the context of critical care, where BBM patients often present with complex, rapidly evolving organ dysfunction and limited physiological reserve, this study offers a clinically grounded approach for early mortality risk stratification. By harnessing first-day ICU data, TabNet captures subtle indicators of systemic collapse, such as SOFA score, hypocalcemia, and hypoalbuminemia, before overt deterioration occurs. Its interpretability and validated generalizability enable informed decisions around ICU triage, prognostic communication, and timely initiation of supportive or goal-concordant care in BBM-associated critical illness.

## Figures and Tables

**Table 1:** Demographic data and baseline characteristics of the MIMIC-IV Database.

Variable	Death Group (N = 266)	Survival Group (N = 599)	*p*-Value
Age [yr, median (IQR)]	67.21 (19.21)	65.19 (17.59)	0.018 *
Height [cm, median (IQR)]	169.50 (21.00)	170.00 (20.00)	0.065
Weight [Kg, median (IQR)]	70.80 (23.11)	76.80 (25.30)	<0.001 *
Gender [N (%)]			
Female	109 (41.0%)	240 (40.1%)	0.822
Male	157 (59.0%)	359 (59.9%)	
Race [N (%)]			
White	200 (75.2%)	450 (75.1%)	0.091
Black	34 (12.8%)	54 (9.0%)	
Asian	13 (4.9%)	29 (4.8%)	
Other	9 (3.4%)	46 (7.7%)	
Unknown	10 (3.8%)	20 (3.3%)	
Comorbidities [N (%)]			
CHF	42 (15.8%)	91 (15.2%)	0.902
CPD	63 (23.7%)	136 (22.7%)	0.819
Diabetes	50 (18.8%)	121 (20.2%)	0.450
Renal Disease	44 (16.5%)	81 (13.5%)	0.289
Rating System [median (IQR)]			
APACHE II	18.00 (10.00)	14.00 (7.00)	<0.001 *
APS III	41.50 (27.00)	41.00 (24.00)	0.574
CCI	9.00 (3.00)	8.00 (3.00)	<0.001 *
GCS	14.00 (2.75)	15.00 (1.00)	<0.001 *
LODS	3.00 (4.00)	4.00 (4.00)	0.852
SOFA	5.00 (5.00)	2.00 (3.00)	<0.001 *
Vital Signs [median (IQR)]			
SBP (mmHg)	86.00 (20.00)	91.00 (21.00)	<0.001 *
DBP (mmHg)	47.00 (15.00)	47.00 (14.00)	0.693
MBP (mmHg)	57.00 (16.00)	60.00 (15.00)	0.004 *
Heart Rate (beats/min)	117.00 (26.75)	104.00 (30.00)	<0.001 *
Resp Rate (breaths/min)	29.00 (9.00)	26.00 (7.00)	<0.001 *
Temperature (°C)	37.11 (0.77)	37.17 (0.78)	0.048 *
Blood Gas [median (IQR)]			
SpO_2_ (%)	91.00 (6.00)	93.00 (4.00)	<0.001 *
SaO_2_ (%)	94.00 (7.00)	97.00 (5.00)	<0.001 *
PaO_2_ (mmHg)	144.00 (142.50)	182.00 (168.00)	0.004 *
PaCO_2_ (mmHg)	41.00 (16.00)	36.00 (12.00)	0.016 *
Base Excess	−1.00 (7.00)	0.00 (4.00)	0.011 *
Anion Gap (mmol/L)	17.00 (6.00)	15.00 (4.00)	<0.001 *
Bicarbonate (mmol/L)	22.00 (7.00)	23.00 (5.00)	0.001 *
Laboratory Tests [median (IQR)]			
Lactate (mmol/L)	1.70 (1.77)	1.40 (0.90)	<0.001 *
PH	7.37 (0.15)	7.38 (0.09)	<0.001 *
Hematocrit (%)	32.00 (12.00)	34.00 (10.00)	<0.001 *
Hemoglobin (g/dL)	10.55 (3.95)	11.40 (3.30)	<0.001 *
Platelets (10^9^/L)	185.50 (181.25)	218.00 (133.00)	0.002 *
WBC (10^9^/L)	9.50 (8.15)	8.40 (6.30)	0.040 *
INR	1.30 (0.30)	1.20 (0.20)	<0.001 *
APTT (s)	30.10 (8.20)	27.90 (7.05)	<0.001 *
PT (s)	14.55 (3.50)	13.40 (2.20)	<0.001 *
Fibrinogen (mg/dL)	533.00 (514.75)	462.00 (546.00)	0.105
Ionized Calcium (mmol/L)	1.16 (0.22)	1.10 (0.17)	<0.001 *
Chloride (mmol/L)	103.00 (12.00)	102.00 (8.00)	0.328
Potassium (mmol/L)	3.90 (1.20)	4.20 (1.10)	<0.001 *
Sodium (mmol/L)	133.00 (7.00)	135.00 (7.00)	0.002 *
ALT (U/L)	27.00 (43.50)	24.00 (52.00)	0.398
AST (U/L)	52.50 (118.50)	37.00 (65.50)	<0.001 *
ALP (U/L)	140.50 (195.75)	115.00 (120.00)	<0.001 *
Albumin (g/dL)	2.80 (0.88)	3.20 (0.90)	<0.001 *
Glucose (mg/dL)	138.50 (73.50)	149.00 (65.00)	0.103
CK (U/L)	287.50 (558.75)	140.00 (443.00)	<0.001 *
CK-MB (U/L)	4.00 (8.75)	4.00 (11.00)	0.277
LDH (U/L)	483.00 (781.50)	334.00 (515.50)	<0.001 *
Scr Baseline (mg/dL)	0.61 (0.40)	0.60 (0.40)	0.327
Creatinine (mg/dL)	1.10 (1.00)	0.90 (0.60)	0.030 *
BUN (mg/dL)	26.00 (24.00)	19.00 (13.00)	<0.001 *

* means Significance; CHF: Congestive Heart Failure; CPD: Chronic Pulmonary Disease; APACHE II: Acute Physiology And Chronic Health Evaluation II; APS III: Acute Physiology Score III; CCI: Charlson Comorbidity Index; GCS: Glasgow Coma Scale; LODS: Logistic Organ Dysfunction System; SOFA: Sequential Organ Failure Assessment; SBP: Systolic Blood Pressure; DBP: Diastolic Blood Pressure; MBP: Mean Blood Pressure; WBC: White Blood Cell count; INR: International Normalized Ratio; APTT: Activated Partial Thromboplastin Time; PT: Prothrombin Time; ALT: Alanine Aminotransferase; AST: Aspartate Aminotransferase; ALP: Alkaline Phosphatase; CK: Creatine Kinase; CK-MB: Creatine Kinase-Myocardial Band; LDH: Lactate Dehydrogenase; Scr: Serum Creatinine; BUN: Blood Urea Nitrogen.

**Table 2:** Logistic regression analysis of features predicting prognosis.

Variable	Coefficient (β)	OR	Z-Value	95% CI	*p*-Value
Weight	0.017	1.017	3.909	1.008–1.025	<0.001
CCI	−0.231	0.793	−6.677	0.741–0.849	<0.001
SOFA	−0.221	0.802	−7.733	0.758–0.848	<0.001
Heart Rate	−0.018	0.982	−4.637	0.975–0.990	<0.001
Resp Rate	−0.033	0.968	−2.839	0.946–0.990	0.005
Lactate	−0.164	0.849	−3.22	0.768–0.938	0.001
Hematocrit	0.038	1.038	3.061	1.014–1.064	0.002
Calcium	−4.162	0.016	−8.364	0.006–0.041	<0.001
Potassium	0.513	1.671	4.827	1.357–2.058	<0.001
WBC	−0.062	0.940	−5.109	0.918–0.963	<0.001
Albumin	0.253	2.115	4.472	1.523–2.936	<0.001

## Data Availability

The MIMIC-IV (version 3.1) and eICU Collaborative Research Database (version 2.0) are publicly available through the PhysioNet repository (https://physionet.org/) upon credentialed access approval. The clinical dataset from the First Affiliated Hospital of Xinjiang Medical University is not publicly available due to institutional and patient privacy regulations but may be made available by the corresponding author upon reasonable request and with appropriate ethical approval. Model-related code and resources are available on GitHub at https://github.com/Numwyx/streamlit-tabnet-app (accessed on 4 April 2025) and https://github.com/Numwyx/Bone-and-Bone-Marrow-Metastasis-Model-Code (accessed on 9 September 2025).

## Supplementary Materials

The following supporting information can be downloaded at: https://www.mdpi.com/article/10.3390/curroncol32100533/s1, Table S1: Demographic data and baseline characteristics of patients from the regional external validation cohort (eICU-CRD); Table S2: Demographic data and baseline characteristics of patients from the cross-regional external validation cohort (First Affiliated Hospital of Xinjiang Medical University); Table S3: Hyperparameters of Deep Learning models; Supplementary Material S1; Supplementary Material S2; Supplementary Material S3; TRIPOD-AI Checklist. References [86,87] are cited in the Supplementary Materials.

## Abbreviations

The following abbreviations are used in this manuscript:

AP	Average Precision
APACHE II	Acute Physiology and Chronic Health Evaluation II
APS III	Acute Physiology Score III
AUC	Area Under the Curve
BBM	Bone and Bone Marrow Metastasis
BUN	Blood Urea Nitrogen
CCI	Charlson Comorbidity Index
CK	Creatine Kinase
CNN	Convolutional Neural Network
CRD	Collaborative Research Database
DBP	Diastolic Blood Pressure
DL	Deep Learning
DLR	Deep Logistic Regression
eICU	eICU Collaborative Research Database
F1	F1 Score (harmonic mean of precision and recall)
GAN	Generative Adversarial Network
GAM-NN	Generalized Additive Model Neural Network
GCS	Glasgow Coma Scale
GNN	Graph Neural Network
ICU	Intensive Care Unit
IQR	Interquartile Range
LDH	Lactate Dehydrogenase
LODS	Logistic Organ Dysfunction Score
MBP	Mean Blood Pressure
MICE	Multiple Imputation by Chained Equations
MIMIC-IV	Medical Information Mart for Intensive Care IV
MLP	Multilayer Perceptron
PR	Precision–Recall
PT	Prothrombin Time
QNN	Quantum Neural Network
ROC	Receiver Operating Characteristic
SBP	Systolic Blood Pressure
SHAP	SHapley Additive exPlanations
SOFA	Sequential Organ Failure Assessment
TabNet	Tabular Neural Network with Attention Mechanism
TRIPOD	Transparent Reporting of a Multivariable Prediction Model for Individual Prognosis or Diagnosis
WBC	White Blood Cell count
