# Supplementary material for: Deep Learning-Based 30-Day Mortality Prediction in Critically Ill Bone and Bone Marrow Metastasis Patients: A Multicenter Retrospective Cohort Study
Source: Curr Oncol. 2025 Sep 24;32(10):533. doi: 10.3390/curroncol32100533 (PMC12564370; doi:10.3390/curroncol32100533)
Supplement: Supplementary file 1 [file curroncol-32-00533-s001.zip › Supplementary Table S3.pdf]

Supplementary Table S3. Hyperparameters of Deep Learning models.

Models	Hyperparameters
CNN	KerasCNN(input_shape=(28,28,1), conv1_filters=32, conv1_kernel=3, conv1_activation='relu', conv1_padding='same', conv1_regularizer=L2(0.0003), batch_norm=True, pool1_size=2, dropout1=0.30, conv2_filters=64, conv2_kernel=3, conv2_activation='relu', conv2_padding='same', conv2_regularizer=L2(0.0003), batch_norm=True, pool2_size=2, dropout2=0.30, flatten=True, dense_units=128, dense_activation='relu', dense_regularizer=L2(0.0003), dropout_dense=0.30, output_units=1, output_activation='sigmoid', optimizer=Adam(lr=0.001), loss='binary_crossentropy', metrics='AUC', batch_size=64, epochs=60, early_stopping(monitor='val_auc', mode='max', patience=10, restore_best_weights=True), validation_split=0.20)
Tabnet	TabNetClassifier(n_d=24, n_a=24, n_steps=3, gamma=1.3, lambda_sparse=1e-4, dropout=0.30, mask_type='sparsemax', optimizer=Adam(lr=0.001, weight_decay=0.0003), scheduler=StepLR(step_size=15, gamma=0.9), max_epochs=100, patience=20, batch_size=256, virtual_batch_size=64, num_workers=0, drop_last=False, eval_metric='auc', class_weights=inv_freq_per_class)
Deep-GLM	DeepGLM(backend='keras', input_dim=p, dnn_units=(512,256,128), activations='relu', kernel_initializer='he_uniform', batch_norm_layers=(1,2), dropouts=(0.5,0.4,0.3), linear_path=True, combine='add', output_activation='sigmoid', l2_lambda=0.0002, optimizer=Adam(learning_rate=ExponentialDecay(initial_lr=0.0005, decay_steps=1000, decay_rate=0.96, staircase=True)), loss='binary_crossentropy', metrics='AUC', epochs=200, batch_size=64, early_stopping(patience=15, restore_best_weights=True), validation_split=0.15, cv_folds=5, imputation='missForest')
H2O DeepLearning	H2ODeepLearning(activation='RectifierWithDropout', hidden=[64,32], epochs=200, rate=0.001, l2=0.0001, input_dropout_ratio=0.0, hidden_dropout_ratios=[0.5,0.5], balance_classes=True, seed=123, stopping_metric='AUC', stopping_rounds=5, stopping_tolerance=0.001, variable_importances=True, selection=Grid(Cartesian,nfolds=5,fold_assignment='Stratified'))
DLR	DLR(backend='keras-tensorflow', input_dim=p, layers=[64,32], activations='relu', output_activation='sigmoid', l2=0.0003, dropout=[0.30,0.20], optimizer=Adam(lr=0.001), loss='binary_crossentropy', metric='AUC', epochs=50, batch_size=32, early_stopping(monitor='val_auc', mode='max', patience=10, restore_best_weights=True), validation_split=0.15)
GAM-NN	DeepRegressionGAMNN(family='bernoulli', link='logit', additive_formula='~ s(all_predictors)', s_basis='tp', optimizer=Adam(lr=0.001), epochs=100, batch_size=64)
GNN	GNN_GCN(backend='torch', graph_build='cosine→ReLU+self_loop→sym_norm(D^-1/2 S D^-1/2)', input=zscore(train_mean,train_sd), layers=[Linear(p→64), ReLU, Dropout(0.30), Linear(64→2)], message_passing='A·H per layer', hidden=64, dropout=0.30, optimizer=Adam(lr=0.001, weight_decay=0.0003), loss='cross_entropy', metric='AUC', epochs=100, early_stopping(monitor='val_auc', patience=10, restore_best_weights=True), train_val_split='per-fold indices', inference_prob='softmax[:,2]')

MLP	MLP(backend='keras-tensorflow', preprocessing='dummyVars(fullRank=True)+medianImpute+center+scale', input_dim=p, architecture=[128,64,32], activations='relu', batch_norm=EveryHidden, dropout=0.30, l2=0.0003, output_activation='sigmoid', optimizer=Adam(lr=0.001), loss='binary_crossentropy', metric='AUC', epochs=100, batch_size=64, early_stopping(monitor='val_auc', mode='max', patience=20, restore_best_weights=True), reduce_lr_on_plateau(monitor='val_auc', mode='max', factor=0.5, patience=8, min_lr=1e-6), validation_split=0.15)
QNN	QNNVQC(backend='pennylane+torch', n_qubits=p, reps=2, n_layers=2, scale=1.00, lr=0.005, batch_size=64, epochs=60, patience=10, feature_map='AngleEmbedding', ansatz='Rot+RingCNOT', readout='expval(Z0)', optimizer='Adam', loss='BCE', standardize=True, cv='StratifiedKFold(5)', selection='AUC')
Deep-Kernel	GPNeuralKernel(backend='torch+gpytorch', feature_extractor=[Linear(p→128),ReLU,Dropout(0.20),Linear(128→64)], inducing_points=256, kernel='Scale(RBF, ARD=64)', likelihood='Bernoulli', variational='Cholesky; learn_Z=True', optimizer=Adam(lr=0.0005, weight_decay=0.0002), training='VI-ELBO', epochs=60, batch_size=64, early_stopping(monitor='val_auc', patience=10, restore_best=True), scaler='StandardScaler')
GAN	SSGAN_FM(backend='keras-tensorflow', preprocessing='zscore(center,scale)+pad_to_784→reshape_28×28×1', conditional='label_map(28×28×1) concat', generator='latent_dim=64,label_emb=8,Dense(6272)→Reshape(7,7,128)→ConvT(64,5,s=2,ReLU)→ConvT(1,5,s=2,tanh)', discriminator='Conv(64,5,s=2,ReLU)→Dropout(0.30)→Conv(128,5,s=2,ReLU)→Dropout(0.30)→Dense(128,ReLU,L2=0.0003)', disc_output='softmax[real0,real1,fake]', optimizers='D:Adam(lr=0.0002,β1=0.5); G:Adam(lr=0.0002,β1=0.5)', training='adversarial+feature_matching', epochs=60, batch_size=64, final_hparams={dropout=0.30,l2=0.0003})
TransFormer	FT_Transformer(backend='keras-tensorflow', input_dim=p, n_tokens=8, d_model=64, n_blocks=2, n_heads=4, key_dim=16, activation='gelu', dropout=0.30, l2=0.0003, token_mlp='Dense(n_tokens*d_model)→BatchNorm→Dropout→Reshape(n_tokens,d_model)', transformer_block='[MHA(num_heads=4,key_dim=16)→Dropout→Add→LayerNorm]×1 → [FFN(d_model*4→d_model,L2=0.0003,Dropout=0.30)→Add→LayerNorm]×1', pooling='GlobalAveragePooling1D', output='Dense(1,sigmoid)', optimizer=Adam(lr=0.001), loss='binary_crossentropy', metric='AUC', epochs=60, batch_size=64, early_stopping(monitor='val_auc', mode='max', patience=10, restore_best_weights=True), validation_split=0.20)
Triplet-Network	MetricLearning_TripletKNN(backend='keras-tensorflow+FNN', preprocessing='center+scale', base_network='Dense(64,ReLU,L2=0.0003)→Dropout(0.30)→Dense(32,L2=0.0003)', embedding_dim=32, triplet_loss_margin=0.20, optimizer=Adam(lr=0.001), epochs=25, batch_size=32, num_triplets=5000, kNN_k=5)
