# Supplementary material for: Deep Learning-Based 30-Day Mortality Prediction in Critically Ill Bone and Bone Marrow Metastasis Patients: A Multicenter Retrospective Cohort Study
Source: Curr Oncol. 2025 Sep 24;32(10):533. doi: 10.3390/curroncol32100533 (PMC12564370; doi:10.3390/curroncol32100533)
Supplement: Supplementary file 1 [file curroncol-32-00533-s001.zip › Supplementary Material S1.pdf]

Supplementary Material S1

1. MLP (Feed-forward Neural Network)

A regularized feed-forward neural network was implemented for binary risk prediction. To minimize information leakage, preprocessing was re-estimated within each cross-validation fold: categorical variables were one-hot encoded with full rank (caret dummyVars), and continuous predictors were centered and scaled (preProcess). The architecture comprised two to three fully connected hidden layers with rectified linear unit activations, each followed by batch normalization and dropout, and terminated with a single sigmoid output. Regularization combined dropout and L2 weight decay. Training employed the Adam optimizer with binary cross-entropy loss, and performance was monitored using the area under the receiver operating characteristic curve (AUC). Hyperparameters were tuned by stratified five-fold cross-validation across a grid spanning layer widths {64,32}, {128,64,32}, and {64,64,32}; dropout rates {0.2, 0.3}; L2 penalties $\{1\times10^{-4}, 3\times10^{-4}\}$; learning rates $\{1\times10^{-3}, 3\times10^{-4}\}$; and batch sizes {32, 64}, with training capped at 100 epochs.

2. CNN (LeNet-5-Style)

A LeNet-5-style two-dimensional convolutional neural network (CNN) was implemented for binary risk prediction. Predictors were z-score standardized on the training set (means and scales reused for the validation cohort), then zero-padded to 784 dimensions and reshaped into single-channel 28×28 "pseudo-images." The architecture comprised two convolutional blocks—each Conv2D(3×3, 32/64 filters) → batch normalization → ReLU → max-pooling (2×2) → dropout—followed by Flatten → Dense(128, ReLU) → Dropout → Dense(1, Sigmoid). L2 weight decay and dropout provided regularization. Training used the Adam optimizer with binary cross-entropy loss; performance was monitored by the AUC. Hyperparameters were tuned by stratified five-fold cross-validation over dropout {0.2, 0.3}, L2 penalties $\{1\times10^{-4}, 3\times10^{-4}\}$, and learning rates $\{1\times10^{-3}, 5\times10^{-4}\}$, with up to 60 epochs and batch size 64.

3. DeepLearning (H_2O)

A deep feed-forward neural architecture was implemented with flexible hidden layer configurations. Candidate structures comprised either two hidden layers of sizes {64,32} or three hidden layers of sizes {128,64,32}. Each hidden layer employed rectified linear unit (ReLU) activation functions, enabling non-linear transformations across successive layers. To enhance robustness and reduce overfitting, dropout regularization was applied both at the network input (0–0.1) and within the hidden layers, with candidate ratios including both uniform (e.g., 0.5,0.5) and progressively decreasing ratios across layers (e.g., 0.5–0.4–0.3). Weight regularization was further enforced through L2 penalties ranging from 1×10^{-4} to 3×10^{-4}.

4. Deep-GLM

A Deep-GLM architecture was designed to combine the strengths of a linear predictor and a deep nonlinear transformation within a unified network. The model accepted tabular inputs and processed them through two parallel branches. The nonlinear branch consisted of three fully connected hidden layers with progressively decreasing width (512–256–128 or 256–128–64), each employing rectified linear unit (ReLU) activation functions. To improve

stability and mitigate overfitting, each hidden layer was followed by batch normalization and dropout, with dropout ratios decreasing across layers (e.g., 0.5–0.4–0.3). L2 weight regularization was optionally applied to further constrain model complexity. In parallel, the linear branch mapped the same inputs directly to a single linear unit, thereby preserving the structure of a conventional generalized linear model. The outputs from the nonlinear and linear pathways were combined additively at the logit scale and subsequently transformed by a sigmoid activation to produce calibrated probabilities.

5.DLR

A DLR architecture was implemented to extend the representational capacity of classical logistic regression by introducing nonlinear hidden layers. The input features were first mapped into two successive fully connected layers with widths fixed at 64 and 32 units, each activated by rectified linear units (ReLU). To improve stability and reduce overfitting, dropout regularization was applied after each hidden layer, with dropout rates explored over a grid spanning {0.3, 0.2} for the first hidden layer and {0.2, 0.1} for the second. In addition, L2 weight regularization was imposed on all dense layers, with penalties explored between 1×10^{-4} and 3×10^{-4}. The final prediction was generated by a single sigmoid-activated output unit, preserving the probabilistic interpretation of a logistic regression classifier. The optimization process employed the Adam optimizer with learning rates tuned between 5×10^{-4} and 1×10^{-3}. Model training was capped at 50 epochs per run with a batch size of 32.

6. Transformer (Feature Tokenization)

A Transformer-based architecture designed for tabular data was implemented in line with the FT-Transformer paradigm. Prior to model construction, continuous predictors were standardized to zero mean and unit variance using training-set statistics, and the same scaling parameters were applied to the validation and test cohorts to prevent information leakage. The standardized features were then projected through a dense layer into a tokenized representation of fixed length (n_tokens = 8), with each token encoded as a d_model-dimensional vector (64 or 128). Batch normalization followed by dropout (0.2–0.3) was applied immediately after projection and before reshaping into the token sequence, ensuring stability and regularization at the tokenization stage. The encoder comprised n_blocks = 2 stacked Transformer blocks. Each block employed multi-head self-attention (n_heads = 4, with key_dim = d_model / n_heads) to model dependencies among feature tokens. Outputs from the attention layer underwent dropout and were integrated with the residual stream, followed by post-norm layer normalization, ensuring stable gradient flow. The feed-forward sublayer expanded each token to width 4 × d_model, applied GELU activation, then contracted it back to d_model; dropout and L2 penalties (1×10^{-4}–3×10^{-4}) were applied at each transformation. A second residual connection with post-norm layer normalization completed the block. After the encoder stack, token embeddings were aggregated via global average pooling across the sequence dimension, producing a compact representation of the feature set without the use of a [CLS] token. This pooled vector was further refined by a dense layer of width d_model with ReLU activation and dropout, and finally mapped to binary probabilities through a single sigmoid output unit. Regularization combined dropout at multiple stages (tokenization, attention, feed-forward, and dense head) with L2 penalties on kernel weights. Optimization used Adam with learning rates in the range of 5×10^{-4}–1×10^{-3}, binary cross-entropy loss, and early stopping (patience = 10) based on validation AUC.

7. TabNet (Supervised)

A supervised TabNet classifier was implemented, following the standard architecture with sparsemax feature selection masks and multiple decision steps. Input features were one-hot encoded with full rank, centered, and scaled within each cross-validation fold to avoid information leakage. Each instance was transformed into a sequence of tokens through the feature transformer, where fully connected layers projected features into shared embeddings. At each decision step, the attentive transformer applied sparsemax masks to adaptively select subsets of features, while residual connections ensured stability and information flow. Feature transformers within the steps used independent and shared layers to balance global and local representation learning. The model dimension was controlled by the feature and attentive widths (n_d = n_a = 24), with three decision steps (n_steps = 3) and $\gamma = 1.3$ controlling the relaxation of mask sparsity. Dropout was applied at multiple stages (0.2–0.3), combined with L2 weight penalties (1×10^{-4}–3×10^{-4}) on all linear projections for regularization. The Adam optimizer was used with learning rates ranging from 1×10^{-3} to 1×10^{-2}, weight decay matched to the L2 penalty, and StepLR scheduling (decay factor 0.9 every 15 epochs). Training was capped at 100 epochs with early stopping (patience = 20, best-weight restoration), batch size 256, and virtual batch size 64.

8. GAM-NN

The GAM-NN was implemented using the deepregression framework, where each predictor was mapped through a feature-specific subnetwork created with the s() function. By default, each subnetwork consisted of a single hidden dense layer with approximately 20 units, nonlinear activation, and no explicit dropout or L2 penalty. The outputs of these subnetworks were summed additively on the logit scale and transformed by a logistic link to yield binary probabilities. Hyperparameters were tuned by stratified five-fold cross-validation across learning rates $\{1\times10^{-2}, 1\times10^{-3}, 5\times10^{-4}, 1\times10^{-4}\}$, epochs {30, 50, 100}, and batch sizes {16, 32, 64, 128}, while architectural parameters such as hidden width, number of layers, and regularization remained fixed at default values, and training proceeded for the specified number of epochs without early stopping.

9. GNN (Kipf–Welling)

A graph convolutional network (GCN) following the Kipf–Welling formulation was implemented to exploit relational structure among samples. Each sample was represented by its tabular features, which were z-score standardized on the training set (means and standard deviations reused for validation data). A fully connected similarity graph was constructed using cosine similarity between feature vectors, thresholded at zero with a ReLU to enforce non-negative affinities, augmented with self-loops, and symmetrically normalized to yield the propagation matrix $\hat{A} = D^{-1/2}(A + I)D^{-1/2}$. The network itself comprised two spectral graph convolution layers: the first projected the input into a hidden representation (64 or 128 units), followed by ReLU activation and dropout (0.2–0.3), and the second mapped to a two-class output. L2 regularization (1×10^{-4}–3×10^{-4}) was applied to kernel weights. Training was conducted with Adam (learning rate 5×10^{-4}–1×10^{-3}) and cross-entropy loss, with early stopping (patience = 10) based on validation AUC to prevent overfitting. Hyperparameters tuned by stratified five-fold cross-validation included hidden dimension, dropout rate, L2 penalty, and learning rate, with the optimal configuration subsequently retrained on the full

training set. Final probabilities were obtained by applying a softmax to the output logits, and model performance was assessed on an independent validation cohort.

10. GAN (Semi-Supervised and Feature Matching)

A semi-supervised, label-conditional GAN with feature matching was implemented using a K+1 discriminator. Tabular predictors were z-score standardized, zero-padded to 784, and reshaped to 28×28×1 pseudo-images; class labels conditioned both networks (generator via an 8-dimensional embedding concatenated with the latent; discriminator via a label map replicated to 28×28 and concatenated as an extra channel). The generator took a 64-dimensional Gaussian latent plus the label embedding and followed Dense(128·7·7) → ReLU → Reshape(7×7×128) → Conv2DTranspose(64, 5×5, stride 2, same, ReLU) → Conv2DTranspose(1, 5×5, stride 2, same, tanh). The discriminator operated on the 2-channel input with Conv2D(64, 5×5, stride 2, same, ReLU) → Dropout → Conv2D(128, 5×5, stride 2, same, ReLU) → Dropout → Flatten → Dense(128, ReLU, name="feat") → Dense(3, Softmax), yielding logits for real-class-0, real-class-1, and fake. Regularization comprised dropout (0.2–0.3) and L2 weight decay (1×10^{-4}–3×10^{-4}) on convolutional/dense kernels. Training alternated discriminator updates (three-way cross-entropy; Adam with β_1=0.5 and learning rate fixed at 2×10^{-4}) with two generator objectives: (i) an adversarial K-class loss in which the conditioning label for generated samples was uniformly sampled from {0,1} (independent of data labels), optimized with Adam(β_1=0.5, lr=2×10^{-4}) while freezing the discriminator; and (ii) feature matching, minimizing MSE between the mean "feat"-layer representation of real and generated batches (Adam, lr=2×10^{-4}). Models were trained for 60 epochs with batch size 64 and no early stopping. Hyperparameters were selected by stratified five-fold cross-validation only over dropout {0.2, 0.3} and L2 {1×10^{-4}, 3×10^{-4}}; although the search grid listed learning rates {1×10^{-3}, 5×10^{-4}}, the implementation kept the learning rate fixed at 2×10^{-4} and thus did not tune it. For evaluation, the discriminator's three-way output was converted to a label-conditioned risk score consistent with the implementation; for label-agnostic inference, the appropriate score is obtained by evaluating the discriminator twice (y=0 and y=1) to compute p_0 and p_1 and returning $p_1/(p_0 + p_1)$.

11. Deep-Kernel (SVGP, ARD-RBF, Bernoulli)

A deep kernel learning (DKL) binary classifier was implemented, coupling a learnable front-end feature extractor with a variational sparse Gaussian process (SVGP) back-end. Inputs were standardized (StandardScaler) and passed to a feed-forward feature network Linear(hidden) → ReLU → Dropout → Linear(out_dim), with hyperparameters searched over hidden ∈ {64, 128}, feat_dim ∈ {32, 64}, and dropout ∈ {0.1, 0.2}. In the resulting embedding space, a GP with ConstantMean and ScaleKernel(RBFKernel) was used, enabling automatic relevance determination via ARD (ard_num_dims = feat_dim). Approximate inference employed VariationalStrategy with a CholeskyVariationalDistribution (SVGP); the set of inducing points (m ∈ {128, 256}) was initialized by forwarding a random subset through the feature net and was learned jointly (learn_inducing_locations = True). The likelihood was Bernoulli, and optimization maximized the variational ELBO using Adam with lr ∈ {1×10^{-3}, 5×10^{-4}} and weight decay ∈ {1×10^{-4}, 2×10^{-4}} under batch size = 64. Model selection used stratified 5-fold cross-validation with early stopping on validation AUC (patience = 7, up to 50 epochs per fold); the selected configuration was then refit on the full

training data with a fixed 15% hold-out subset for monitoring (patience = 10, up to 60 epochs). Final risk scores were the Bernoulli predictive probabilities produced by the GP likelihood in the learned feature space.

12. QNN (Variational Quantum Classifier)

A QNN was implemented as a hybrid quantum–classical binary model. Tabular inputs were z-score standardized and embedded onto p qubits via AngleEmbedding (scale $\in$ {0.5, 1.0}). The parameterized circuit used a hardware-efficient ansatz of per-qubit Rot(θ,φ,ω) gates followed by a ring CNOT entangling pattern, stacked for n_layers $\in$ {1, 2, 3} and optionally data re-uploading with reps $\in$ {1, 2}. Readout was the expectation of Pauli-Z on qubit 0, linearly mapped to a probability. Training optimized circuit parameters with Adam (learning rate $\in \{1\times10^{-2}, 5\times10^{-3}, 1\times10^{-3}\}$) under binary cross-entropy, using mini-batches {32, 64}, up to 60 epochs, and early stopping (patience = 10) based on validation AUC. Model selection adopted stratified 5-fold cross-validation over the grid {reps, n_layers, scale, lr, batch size}; the chosen configuration was then refit on the full training data with a fixed 15% hold-out subset used solely for callback monitoring. Implementation details: PennyLane default.qubit (analytic, shots=None), parameter-shift differentiation, and probabilities produced directly from the measured expectation values.

13. Triplet Network (Siamese, Margin Triplet Loss)

A Triplet Network was implemented for metric learning, in which three weight-sharing subnetworks map anchor, positive, and negative samples into a common embedding space. Each subnetwork consisted of a feed-forward encoder (Dense(64, ReLU) → Dropout(0.2–0.3) → Dense(32, linear), with L2 regularization of 1×10^{-4}–3×10^{-4}) that projected inputs into 32-dimensional embeddings. Training used the margin-based triplet loss ($\alpha = 0.2$) to enforce smaller distances between anchor–positive pairs and larger distances for anchor–negative pairs, with embeddings optimized via Adam (lr $\in \{1\times10^{-3}, 5\times10^{-4}\}$). Stratified 5-fold cross-validation guided hyperparameter selection over dropout, L2 penalties, and learning rates. Final predictions were obtained by computing embeddings of all samples and applying a 5-nearest neighbor classifier in the learned embedding space, where risk scores corresponded to the fraction of neighbors labeled positive.
