# Supplementary material for: Deep Learning-Based 30-Day Mortality Prediction in Critically Ill Bone and Bone Marrow Metastasis Patients: A Multicenter Retrospective Cohort Study
Source: Curr Oncol. 2025 Sep 24;32(10):533. doi: 10.3390/curroncol32100533 (PMC12564370; doi:10.3390/curroncol32100533)
Supplement: Supplementary file 1 [file curroncol-32-00533-s001.zip › Supplementary Material S2.pdf]

Supplementary Material S2

Prior to model comparison, all traditional machine learning algorithms were developed following the same rigorous framework as applied to the deep learning models. A unified preprocessing pipeline was implemented to ensure consistency and to prevent data leakage, after which stratified five-fold cross-validation was conducted for every algorithm. Within this framework, systematic hyperparameter tuning was performed for each model, and the optimized configurations are summarized in the table. The traditional models evaluated included elastic-net logistic regression, random forest, support vector machine with a radial basis kernel, k-nearest neighbors, gradient boosting machine, AdaBoost, XGBoost, LightGBM, and CatBoost.

Hyperparameters of Tarditional Machine Learning Models

Models	Hyperparameters
XGBoost	XGBoost(booster='gbtree', max_depth=6, eta=0.05, subsample=0.8, colsample_bytree=0.8, min_child_weight=5, gamma=0.1, lambda=1.0, alpha=0.0, objective='binary:logistic', eval_metric='auc', nrounds=300, early_stopping_rounds=20, learning_rate=0.05, tree_method='hist')
LightGBM	LightGBM(boosting='gbdt', num_leaves=31, max_depth=-1, feature_fraction=0.8, bagging_fraction=0.8, bagging_freq=5, min_data_in_leaf=20, lambda_l1=0.0, lambda_l2=0.1, objective='binary', metric='auc', learning_rate=0.05, num_boost_round=500, early_stopping_rounds=30)
CatBoost	CatBoost(iterations=500, learning_rate=0.05, depth=6, l2_leaf_reg=3.0, border_count=128, subsample=0.8, loss_function='Logloss', eval_metric='AUC', od_type='Iter', od_wait=30, bootstrap_type='Bayesian', early_stopping_rounds=30)
GB	GB(distribution='bernoulli', n.trees=300, interaction.depth=5, shrinkage=0.05, bag.fraction=0.8, train.fraction=0.8, n.minobsinnode=10, cv.folds=5, keep.data=TRUE, metric='AUC')
AdaBoost	AdaBoost(base_estimator='DecisionTree(max_depth=3)', n_estimators=300, learning_rate=0.05, algorithm='SAMME.R', random_state=123, eval_metric='AUC', cv=5)
LR	LogisticRegression(penalty='elasticnet', solver='saga', C=1.0, l1_ratio=0.5, max_iter=1000, class_weight='balanced', scoring='roc_auc', cv=5)
RF	RandomForest(n_estimators=500, max_depth=None, min_samples_split=5, min_samples_leaf=2, max_features='sqrt', bootstrap=TRUE, criterion='gini', oob_score=TRUE, random_state=123, scoring='roc_auc')
SVM	SVM(kernel='rbf', C=10, gamma=0.01, probability=TRUE, class_weight='balanced', cache_size=500, tol=1e-4, max_iter=2000, scoring='roc_auc', cv=5)
KNN	KNN(n_neighbors=15, weights='distance', metric='minkowski', p=2, leaf_size=30, algorithm='auto', scoring='roc_auc', cv=5)

Under a consistent data split and a unified preprocessing strategy and select decision thresholds using Youden's J, the traditional machine learning methods exhibited strong fitting but limited generalization. Gradient boosting tree models such as LightGBM, CatBoost, and XGBoost achieved the highest discrimination on the training set, with ROC-AUC typically ranging from 0.81 to 0.82, followed by random forests and classical gradient boosting. In contrast, linear or distance-based baselines, including logistic regression, support vector machines, and k-nearest neighbors, showed clearly lower discrimination, around 0.63 to 0.66. Basically, all models' F1 scores generally occupied the middle to lower tier, and the sensitivity–specificity balance proved highly threshold-dependent, as reflected by stretched radar profiles. On the probability scale, Brier scores in the training set often lay near 0.18 to 0.21, while calibration curves in the medium- to high-risk regions revealed systematic deviations, with some models being overly optimistic or overly conservative. The calibration slope remained close to, but not exactly, one, suggesting residual miscalibration. Decision curve analysis further indicated that positive net benefit was concentrated within a relatively narrow threshold band (approximately 0.30–0.70), beyond which the net benefit rapidly converged toward treating all or treating none. The comparative performance of different models on the training set is shown in Figure 1.

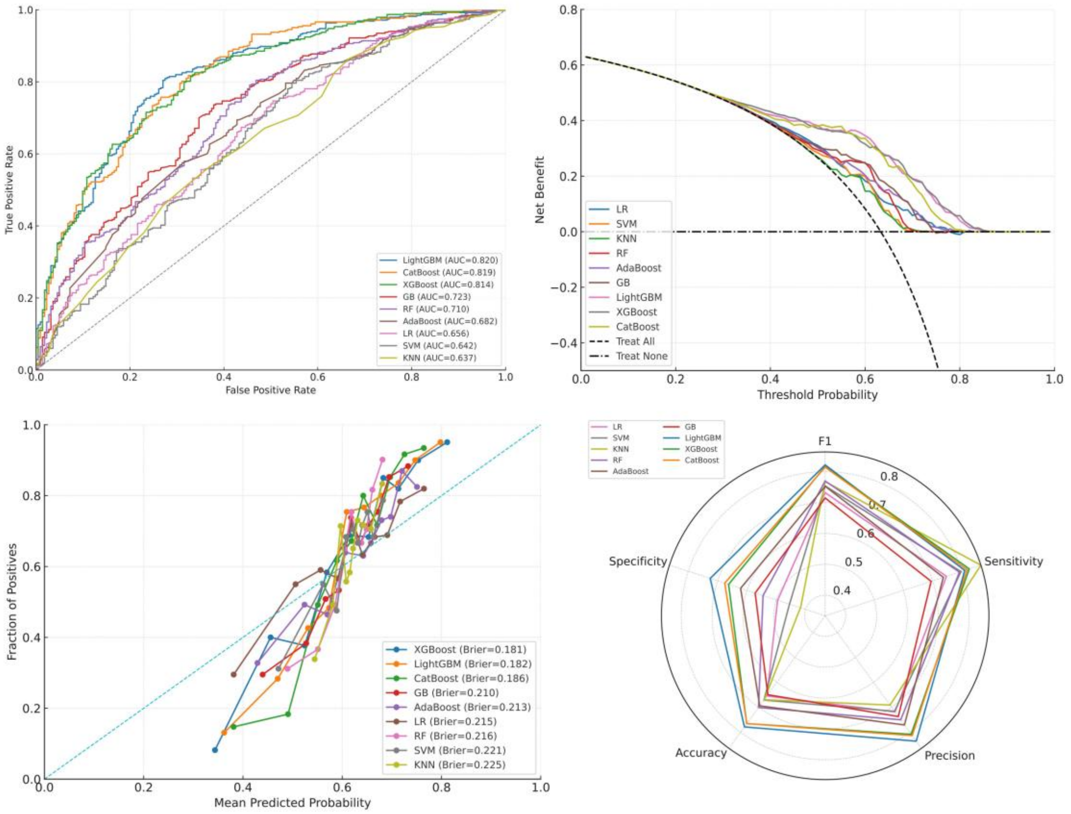

Figure 1 Results of traditional machine learning models on the training set

Top left: ROC curve; Top right: decision curve; Bottom left: calibration curve; Bottom right: non-normalized radar chart.

When moving to the test set almost all models undergo a pronounced drop in discrimination with many ROC-AUC values around 0.50–0.62 and F1 scores falling to about 0.3–0.7 and the sensitivity–specificity trade off deteriorates further so that a gain in sensitivity is often coupled to a marked loss in specificity or the reverse and on calibration the Brier score typically rises to 0.21–0.24+ and points on the calibration curve drift away from the diagonal with slopes tending to less than one and intercepts shifting which reflects probability extrapolation error together with distributional shift and on decision curves most models align with treat all or treat none across a broad span of thresholds or show only marginal advantage so the window of net benefit narrows.

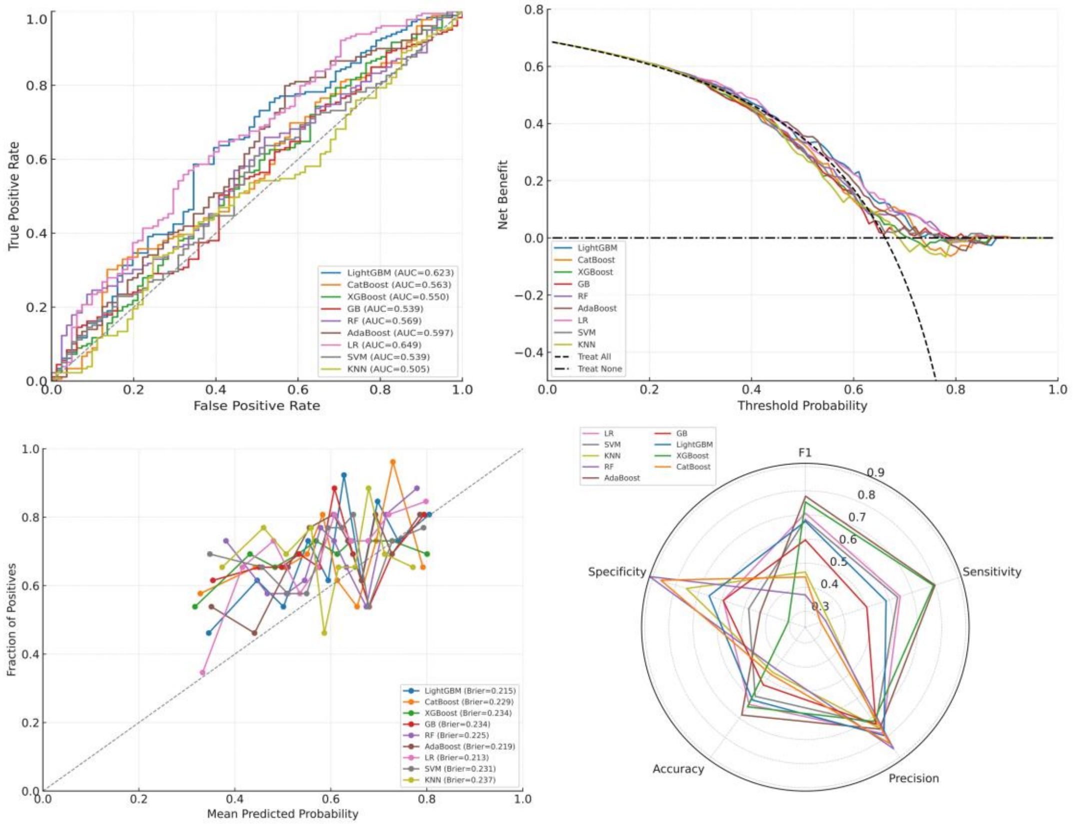

Figure 2 Results of traditional machine learning models on the test set

Top left: ROC curve; Top right: decision curve; Bottom left: calibration curve; Bottom right: non-normalized radar chart.

Taken together the advantages on the training set cannot be maintained on external samples because current predictors are largely single time point or coarse aggregates that fail to encode the genuinely multisystem nonlinear interactions of critical illness and because inter center differences in measurement frequency therapeutic pathways and missingness mechanisms induce distribution shift that prevents correlations learned on the training data from stabilizing into transferable rules therefore models ranging from logistic regression support vector machines and k nearest neighbors to gradient boosting random forests

AdaBoost XGBoost, CatBoost, and LightGBM share the common phenotype of diminished discrimination insufficient calibration and unstable clinical net benefit on both training and test evaluations and precisely for these empirical reasons the study employs deep learning architectures that can represent higher order interactions and thereby aims to deliver more reliable thirty day mortality prediction under real world deployment.
