# Supplementary material for: Deep Learning-Based 30-Day Mortality Prediction in Critically Ill Bone and Bone Marrow Metastasis Patients: A Multicenter Retrospective Cohort Study
Source: Curr Oncol. 2025 Sep 24;32(10):533. doi: 10.3390/curroncol32100533 (PMC12564370; doi:10.3390/curroncol32100533)
Supplement: Supplementary file 1 [file curroncol-32-00533-s001.zip › Supplementary Material S3.pdf]

Supplementary Material S3

1. SOFA

In this study cohort, the overall distribution of SOFA scores in the death group (n = 266) was markedly higher than that in the survival group (n = 599), with the intergroup difference reaching an extremely high level of statistical significance according to the Mann–Whitney U test ($p = 1.30\times10^{-25}$). Point-biserial correlation analysis revealed a significant positive correlation between SOFA scores and mortality risk ($r_{pb} = +0.378$, $p = 8.07\times10^{-31}$), indicating that higher SOFA scores were associated with a higher probability of death. Effect size analysis (Cliff's $\delta = -0.441$) suggested a large practical significance. As shown in the violin plot, the SOFA score distribution in the death group was substantially shifted upward, with both the median and interquartile range clearly higher than those in the survival group, reinforcing the strong association between higher SOFA scores and poor outcomes.

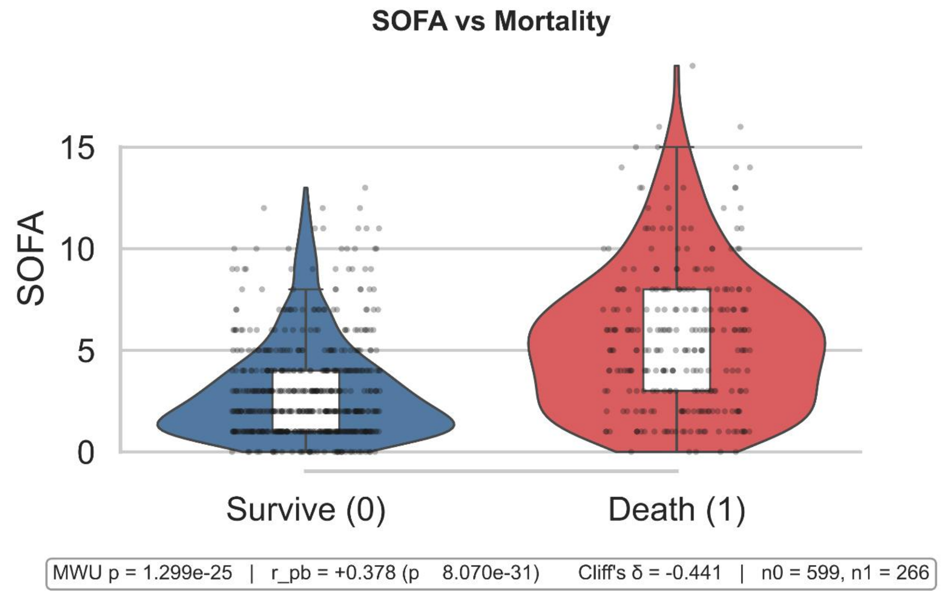

2. Calcium

In this study cohort, the overall distribution of serum calcium levels in the death group (n = 266) was consistently higher than that in the survival group (n = 599), with the intergroup difference reaching statistical significance according to the Mann–Whitney U test ($p = 1.84\times10^{-05}$). Point-biserial correlation analysis demonstrated a significant positive association between calcium levels and mortality ($r_{pb} = +0.136$, $p = 6.12\times10^{-05}$), indicating that elevated calcium was linked to an increased risk of death. Effect size analysis (Cliff's $\delta = -0.182$) indicated a measurable difference between groups. As shown in the violin plot, the calcium distribution in the death group was shifted upward, with both the median and interquartile range higher than those in the survival group, supporting the association between

higher calcium and poorer prognosis.

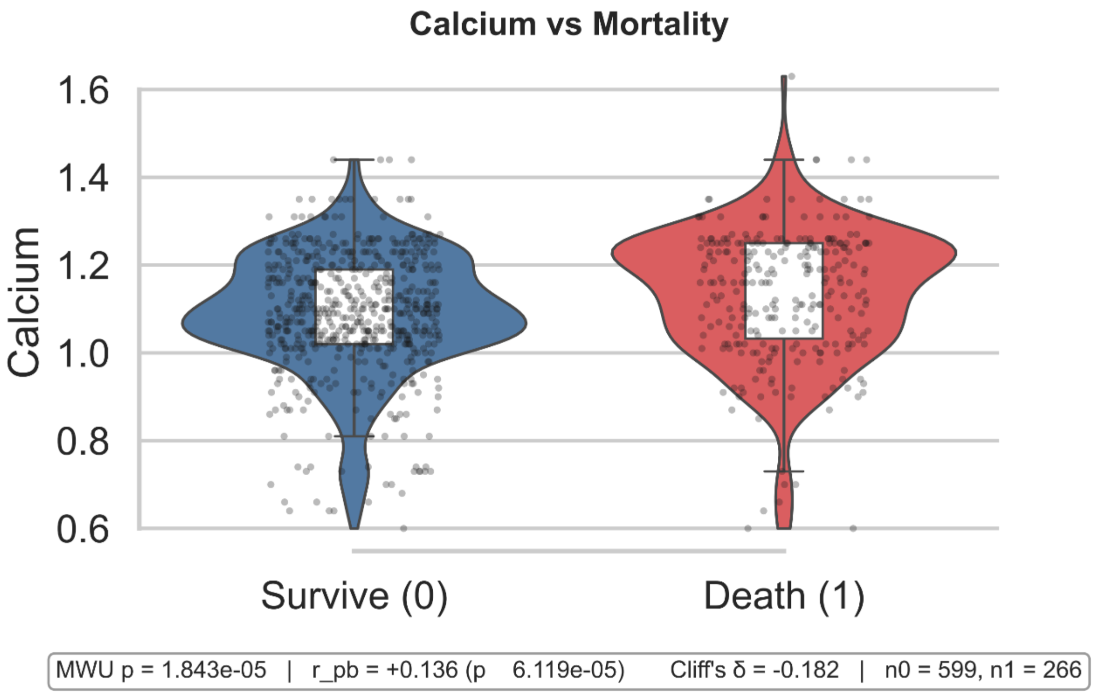

3. Albumin

In this study cohort, the overall distribution of serum albumin levels in the death group (n = 266) was markedly lower than that in the survival group (n = 599), with the intergroup difference reaching a high level of statistical significance according to the Mann–Whitney U test (p = 4.51×10^-17). Point-biserial correlation analysis indicated a significant negative association between albumin levels and mortality (r_pb = −0.287, p = 6.38×10^-18), suggesting that lower albumin levels were associated with a higher probability of death. Effect size analysis (Cliff's δ = +0.357) indicated a moderate practical significance. As shown in the violin plot, the albumin distribution in the death group was shifted downward overall, with both the median and interquartile range lower than those in the survival group, indicating that low albumin levels are not only statistically significant but also closely related to adverse outcomes in terms of overall distribution trends.

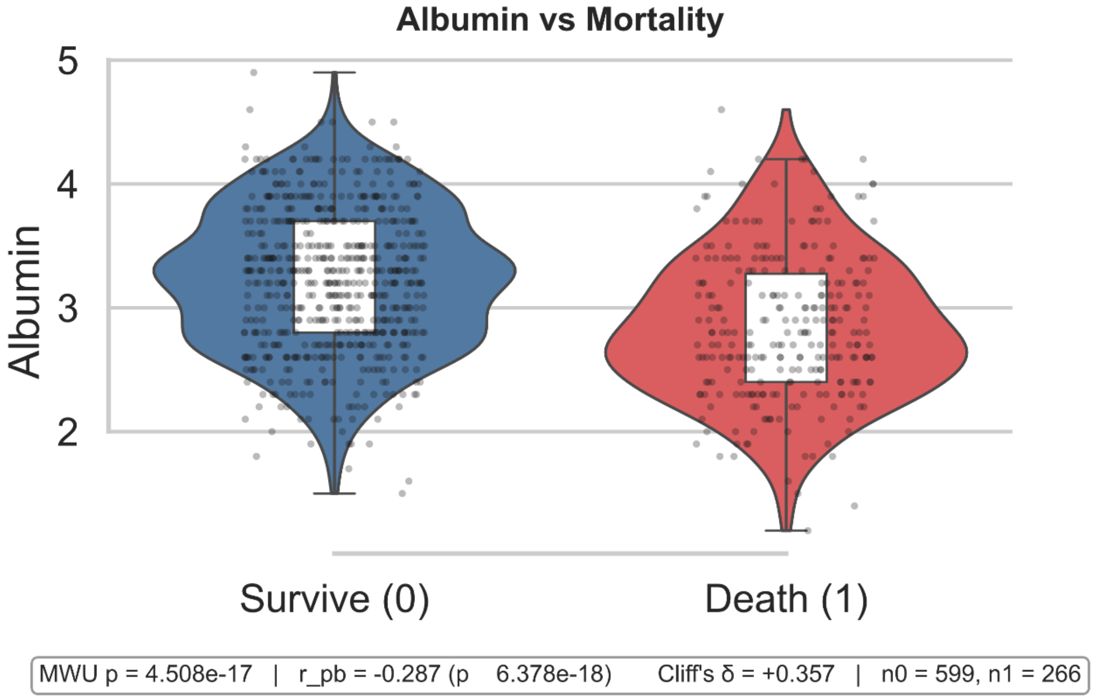

4. Charlson Comorbidity Index (CCI)

In this study cohort, the overall distribution of the Charlson Comorbidity Index in the death group (n = 266) was significantly higher than that in the survival group (n = 599), with the intergroup difference reaching a high level of statistical significance according to the Mann–Whitney U test (p = 2.23×10^-08). Point-biserial correlation analysis revealed a significant positive association between CCI and mortality (r_pb = +0.208, p = 6.76×10^-10), indicating that a greater comorbidity burden was associated with a higher probability of death. Effect size analysis (Cliff's δ = −0.236) indicated a moderate practical significance. The violin plot shows that the distribution of CCI in the death group was shifted upward overall, with higher medians and interquartile ranges compared with the survival group, further supporting the association between greater comorbidity burden and adverse outcomes.

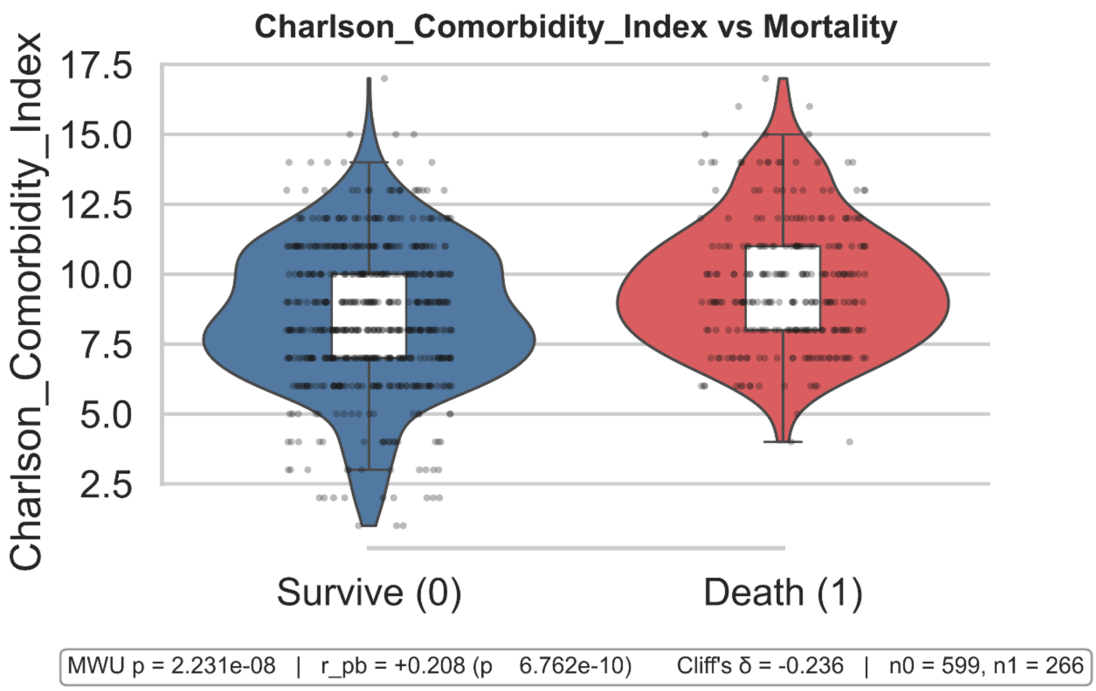

5. Potassium

In this study cohort, the overall distribution of serum potassium levels in the death group (n = 266) was slightly higher than that in the survival group (n = 599), with the intergroup difference reaching statistical significance according to the Mann–Whitney U test (p = 5.54×10^-04). Point-biserial correlation analysis showed a positive association between potassium levels and mortality (r_pb = +0.083, p = 1.50×10^-02), indicating that elevated potassium was linked to an increased likelihood of death. Effect size analysis (Cliff's δ = −0.147) indicated a measurable difference between groups. As illustrated in the violin plot, the potassium distribution in the death group was shifted upward, with a slightly higher median compared to the survival group, although there was substantial overlap between groups, consistent with a relatively weaker association with prognosis.

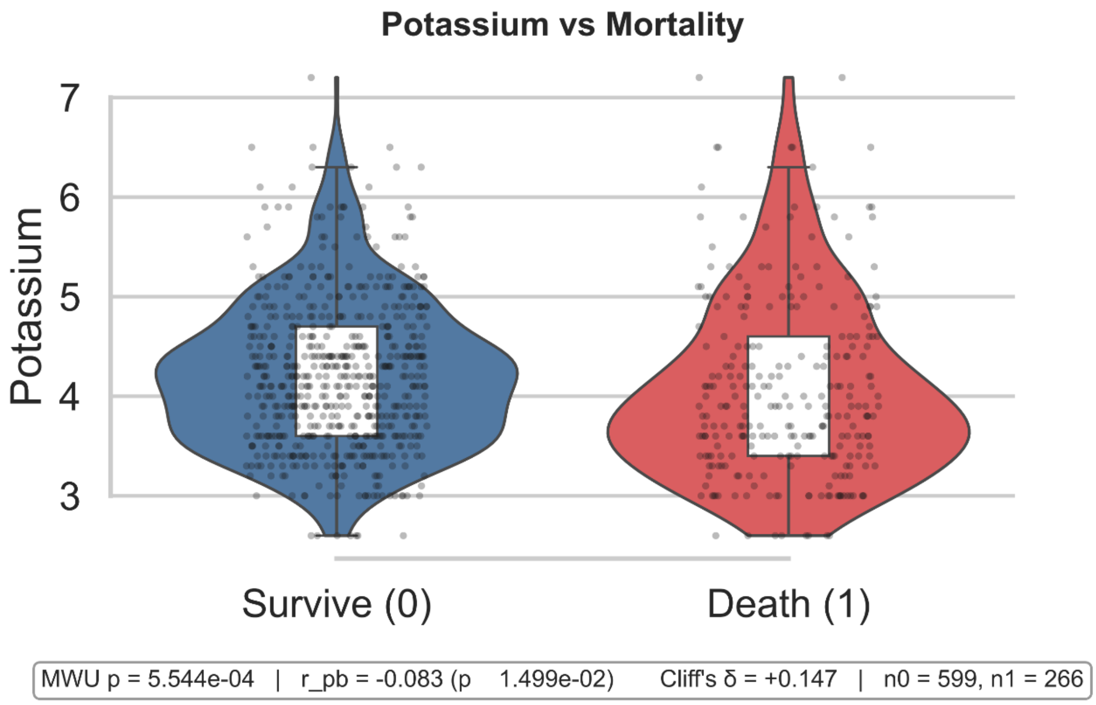
Potassium vs Mortality
Potassium
7
6
5
4
3
Survive (0)
Death (1)
MWU p = 5.544e-04 | r_pb = -0.083 (p 1.499e-02) Cliff's δ = +0.147 | n0 = 599, n1 = 266
