# Supplementary material for: Deep Learning-Based 30-Day Mortality Prediction in Critically Ill Bone and Bone Marrow Metastasis Patients: A Multicenter Retrospective Cohort Study
Source: Curr Oncol. 2025 Sep 24;32(10):533. doi: 10.3390/curroncol32100533 (PMC12564370; doi:10.3390/curroncol32100533)
Supplement: Supplementary file 1 [file curroncol-32-00533-s001.zip › Supplementary Table S1.pdf]

Supplementary Table S1. Demographic data and baseline characteristics of patients from the regional external validation cohort (eICU-CRD).

**Variable [median, IQRs]**	**Survival Group (N=152)**	**Death Group (N=47)**	**P-value**
Weight (kg)	74.05 (27.30)	74.90 (24.50)	0.268
CCI	8.00 (4.00)	8.00 (3.00)	0.472
SOFA	4.00 (3.00)	7.00 (5.50)	<0.001*
Heart Rate (beats/min)	117.00 (28.00)	132.00 (27.00)	<0.001*
Respiratory Rate (breaths/min)	29.00 (10.00)	32.00 (9.00)	0.002*
Lactate (mmol/L)	1.30 (1.19)	1.30 (1.09)	0.710
Hematocrit (%)	33.10 (7.30)	31.30 (7.35)	0.008*
Calcium (mmol/L)	1.12 (0.28)	1.41 (0.26)	<0.001*
Potassium (mmol/L)	4.40 (0.80)	4.90 (1.05)	<0.001*
WBC (10^9/L)	6.37 (5.15)	10.50 (8.75)	0.010*
Albumin (g/dL)	2.40 (0.82)	2.30 (0.60)	0.274
