# Supplementary material for: Deep Learning-Based 30-Day Mortality Prediction in Critically Ill Bone and Bone Marrow Metastasis Patients: A Multicenter Retrospective Cohort Study
Source: Curr Oncol. 2025 Sep 24;32(10):533. doi: 10.3390/curroncol32100533 (PMC12564370; doi:10.3390/curroncol32100533)
Supplement: Supplementary file 1 [file curroncol-32-00533-s001.zip › Supplementary Table S2.pdf]

Supplementary Table S2. Demographic data and baseline characteristics of patients from the cross-regional external validation cohort (First Affiliated Hospital of Xinjiang Medical University).

Variable [median, IQRs]	Survival Group (N=59)	Death Group (N=23)	P-value
Weight (kg)	71.14 (24.18)	69.91 (19.69)	0.804
CCI	8.00 (3.00)	9.00 (2.00)	0.092
SOFA	2.00 (2.50)	6.00 (4.00)	0.001*
Heart Rate (beats/min)	109.00 (35.00)	127.00 (32.00)	0.030*
Respiratory Rate (breaths/min)	25.78 (5.17)	31.00 (6.07)	<0.001*
Lactate (mmol/L)	1.37 (0.76)	1.57 (0.74)	0.157
Hematocrit (%)	34.00 (7.20)	32.00 (7.33)	0.108
Calcium (mmol/L)	1.10 (0.20)	1.27 (0.30)	<0.001*
Potassium (mmol/L)	4.32 (0.67)	4.43 (0.78)	0.635
WBC (10^9/L)	7.98 (6.73)	10.54 (3.48)	0.078
Albumin (g/dL)	2.94 (0.92)	2.68 (0.90)	0.237
